# Supplementary material for: Mechanosensitive non-equilibrium supramolecular polymerization in closed chemical systems
Source: Nat Commun. 2023 May 29;14:3084. doi: 10.1038/s41467-023-38948-x (PMC10227035; doi:10.1038/s41467-023-38948-x)
Supplement: Supplementary file 1 — Supplementary Information [file 41467_2023_38948_MOESM1_ESM.pdf]

Supplementary Information

Mechanosensitive non-equilibrium supramolecular polymerization in closed chemical systems

Xianhua Lang[1#], Yingjie Huang[1#], Lirong He[1], Yixi Wang[2], Udayabhaskararao Thumu[2], Zonglin Chu[3], Wilhelm T. S. Huck[4], Hui Zhao[1*]

[1]School of Chemical Engineering, State Key Lab of Polymer Materials Engineering, Sichuan University, Chengdu 610065, China.

[2]Institute of Fundamental and Frontier Sciences, University of Electronic Science and Technology of China, Chengdu 610054, China.

[3]College of Chemistry and Chemical Engineering, Hunan University, Changsha 410082, China.

[4]Institute for Molecules and Materials, Radboud University, Nijmegen, The Netherlands.

*Corresponding author:

Email:zhaohuichem@scu.edu.cn

[#]These authors contributed equally: Xianhua Lang and Yingjie Huang

Table of Contents

Experimental Procedures 3

1. Instruments. 3
2. Synthesis of compounds 4
3. Preparation of buffer solutions: 7
4. The influence of viologen derivatives on supramolecular self-assembly. 8
5. The influence of pH on supramolecular self-assembly. 9
6. Supramolecular polymerization via charge transfer and amphiphilic interaction. 10
7. Redox kinetics of C_{12}-MV^{2+} investigated by UV-vis measurements. 14
8. Redox kinetics of C_{12}-MV^{2+} investigated by ^{1}H-NMR spectra. 15
9. Redox kinetics of C_{12}-MV^{2+} investigated by EPR measurements. 16
10. The dissipative performance can be activated again by introducing fresh air in a closed system. 17
11. Gas phase changes in redox processes. 18
12. C_{12}-MV^{2+} / PN dissipation states at different times. 19
13. Dissipation states of C_{12}-MV^{2+}/PN induced by phenyl lactate chirality at different times. 21
14. Redox kinetics of C_{12}-MV^{2+}/PN investigated by EPR measurements. 23
15. Redox kinetics of C_{12}-MV^{2+}/PN in the presence of chiral molecules investigated by UV-vis measurements. 24
16. Fluorescence quantum yields 25
17. Fluorescence of PN could be quenched by the CT interaction with C_{12}-MV^{2+}. 26
18. Redox kinetics of MV^{2+}/PN investigated by fluorescence spectroscopy. 27
19. The fluorescence of PN is absorbed by MV^{2+} 28
20. Redox kinetics of MV^{2+} investigated by discoloration. 29
21. Redox kinetics of MV^{2+} investigated by UV-vis measurements. 30
22. Redox kinetics of MV^{2+}/PN investigated by UV-vis measurements. 31
23. Ultrasound-induced patterning. 32
24. Reasons for the patterning induced by ultrasound. 38
25. Ultrasound-induced patterning in frequencies. 43
26. Ultrasound-induced patterning in different molds. 44
27. NMR spectrum. 45

Experimental Procedures

1. Instruments.

Nuclear magnetic resonance (NMR) spectroscopy. NMR spectra were recorded on a JNM-ECZ400S/L1 400 MHz NMR spectrometer. Deuterated dimethyl sulfoxide (DMSO-d_6), deuterated chloroform ($CDCl_3$), and deuterium oxide (D_2O) were used as solvents.

UV-vis spectroscopy. UV absorbance spectra was performed by a SHIMADZU UV1900 UV spectrophotometer.

Fluorescence spectroscopy. Fluorescence spectra were recorded on a HITACHI F-4700 fluorescence spectrophotometer. Excitation wavelength: 320 nm, Slit width: 1nm or 2.5nm, (ex/em): 5/10 nm.

Surface tension. Surface tension was measured using QBZY automatic surface tension meter

Viscosity. The viscosity is measured by Ubbelohde viscometer. The diameter of the capillary is 0.9-1.0 mm. And the viscosity of the solutions was confirmed based on equation (1).

$$\eta = \frac{\pi \rho g h r^4}{8LV} t \qquad (1)$$

η---viscosity; r---capillary radius; L---capillary length; V---capillary volume; g---gravitational acceleration; ρ---solution density; t---liquid flow time through the capillary.

lectron paramagnetic resonance. EPR spectra were obtained using MS-5000X spectrometer.

Dynamic light scattering (DLS). DLS was carried out by a Malvern Zetasizer (Nano ZS90).

Transmission Electron Microscope (TEM). TEM images were recorded on JEOL JEM F200 at 200 kV. The samples were dried under high vacuum for 30 min before TEM test.

Circular Dichroism (CD). The CD spectra were recorded in a Jasco J-810 circular dichroism spectrometer at RT. Spectra were collected at a scan rate of 200 nm•S^{-1} and 2 nm bandwidth with three-times scans for averaging.

Ultrasonic Cleaner. Ultrasonically induced patterning is done by placing the molds into the ultrasound Cleaner of Shanghai Jingqi Instrument Co. Model SB-120DT3L. Power 120w. Frequency 40kHz. And the variable frequency ultrasound cleaner: Fuzou Desen Precision Ltd. DSA50-GL_2-2.5L. Frequency 28kHz/40kHz.

Patterned molds. Round PTFE mold with 7cm diameter and 0.5 cm depth. Positive triangle mold side length 6.7 cm. Square mold side length 6.1 cm.

Gas Chromatography. Huifen GC-7820, with a 5A molecular sieve columns.

Gas detectors. Kallu Electronic. K-100A (N_2, 70%~100%vol). K-400A (O_2, 0.0~30.0%vol, NO, 0~250ppm, NO_2, 0.0~20.0ppm). With portable gas sampling pump.

Low frequency acoustic vibration equipment. Waveform generator: China-victor VICTOR2040H. Loud speaker: COSTE 89mm (Round) 6Ω.

2. Synthesis of compounds

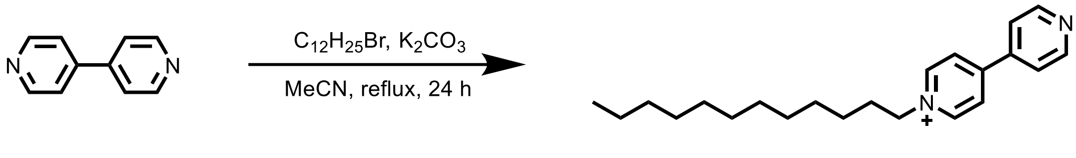

1

Supplementary Figure 1. Synthesis of 1-dodecyl-[4,4'-bipyridin]-1-ium.

In a 200 mL round-bottom flask, 2,2'-Dipyridyl (2.35 g, 15 mmol) and 1-Bromododecane (16.5 mmol, 1.1 eq) were dissolved in 40 mL of MeCN. And then Potassium carbonate (20 mmol) was added to above mixture. The mixture was stirred at 85 °C for 24 h under N_2 atmosphere. After removing all the solids by filtration, the solvent was removed by evaporation. The white solid was obtained after washing with dichloromethane and acetonitrile, and dried in a vacuum oven at 40 °C for 24 h (4.2 g, 70% yield). ^{1}H NMR (400 MHz, DMSO-d_6) δ 9.27 (d, 2H), 8.87 (d, 2H), 8.65 (d, 2H), 8.06 (d, 2H), 4.65 (t, 2H), 1.96 (s, 2H), 1.24 (d, 18H), 0.80 (m, 3H). ^{13}C NMR (101 MHz, DMSO-d_6) δ 152.76, 151.51, 145.85, 141.42, 125.93, 122.47, 60.91, 31.80, 31.25, 29.52, 29.43, 29.31, 29.22, 28.94, 25.93, 22.60, 14.46.

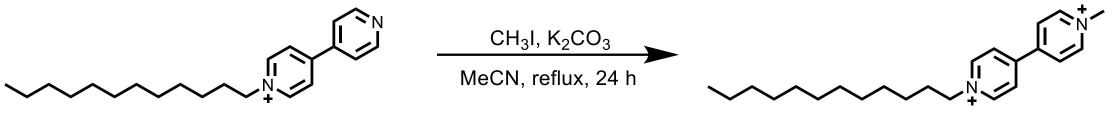

Supplementary Figure 2. Synthesis of 1-dodecyl-1'-methyl-[4,4'-bipyridine]-1,1'-diium (C_{12}-MV^{2+}).

In a 200 mL round-bottom flask, compound 1 (4 g, 9.8 mmol) and CH_3I (27 mmol, 3 eq) were dissolved in 60 mL of MeCN, then the solution was refluxed for 24 h under N_2. The solution was evaporated. After washing with dichloromethane and acetonitrile, the pure C_{12}-MV^{2+} was obtained as a red solid by drying in a vacuum oven at 40 °C for 48 h (4.87 g, 91% yield). ^{1}H NMR (400 MHz, DMSO-d_6) δ 9.40 (d, 2H), 9.29 (s, 2H), 8.79 (dd, 4H), 4.70 (t, 2H), 4.45 (s, 3H), 1.98 (s, 2H), 1.25 (m, 18H), 0.80 (m, 3H). ^{13}C NMR (101 MHz, DMSO-d_6) δ 149.07, 148.73, 147.11, 146.25, 61.43, 48.64, 31.81, 31.29, 29.52, 29.46, 29.34, 29.22, 28.95, 25.95, 22.61, 14.49.

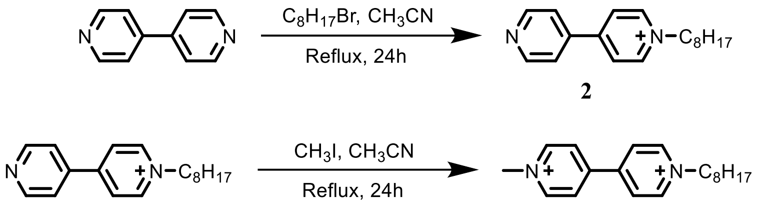

2 **C_8-MV^{2+}**

Supplementary Figure 3. Synthesis of 1-methyl-1'-octyl-[4,4'-bipyridine]-1,1'-diium (C_8-MV^{2+}).

The process of C_8-MV^{2+} synthesis is similar to the preparation of C_{12}-MV^{2+}.
Component 2: White solid，^{1}H NMR (400 MHz, DMSO-d_6) δ 9.33 (d, J = 6.4 Hz, 2H), 8.96 – 8.81 (m, 2H), 8.69 (d, J = 6.6 Hz, 2H), 8.17 – 8.02 (m, 2H), 4.70 (t, J = 7.5 Hz, 2H), 1.97 (p, J = 7.0 Hz, 2H), 1.48 – 1.15 (m, 11H), 0.96 – 0.77 (m, 3H).
C_8-MV^{2+}: Red solid，^{1}H NMR (400 MHz, DMSO-d_6) δ 9.47 – 9.35 (m, 2H), 9.34 – 9.18 (m, 2H), 8.91 – 8.63 (m, 4H), 4.71 (t, J = 7.4 Hz, 2H), 4.46 (s, 3H), 1.98 (h, J = 7.4, 6.8 Hz, 2H), 1.40 – 1.14 (m, 10H), 0.92 – 0.79 (m, 3H).

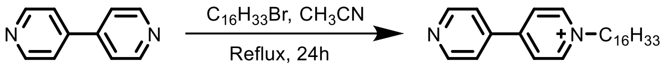

2

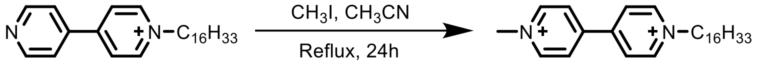

2 **C_{16}-MV^{2+}**

Supplementary Figure 4. Synthesis of 1-hexadecyl-1'-methyl-[4,4'-bipyridine]-1,1'-diium (C_{16}-MV^{2+}).

The synthesis process of C_{16}-MV^{2+} is similar to the preparation process of C_{12}-MV^{2+}.
Compound 3: White solid，^{1}H NMR (400 MHz, DMSO-d_6) δ 9.42 – 9.14 (m, 2H), 8.96 – 8.78 (m, 2H), 8.66 (d, J = 6.8 Hz, 2H), 8.15 – 7.96 (m, 2H), 4.65 (t, J = 7.4 Hz, 2H), 1.97 (q, J = 7.3 Hz, 2H), 1.49 – 1.10 (m, 27H), 0.85 (t, J = 6.7 Hz, 3H).
C_{16}-MV^{2+}: Red solid，^{1}H NMR (400 MHz, DMSO-d_6) δ 9.41 (d, J = 6.8 Hz, 2H), 9.31 (d, J = 6.8 Hz, 2H), 8.80 (dd, J = 10.5, 6.8 Hz, 4H), 4.70 (t, J = 7.4 Hz, 2H), 4.45 (s, 3H), 1.98 (s, 2H), 1.24 (s, 26H), 0.85 (t, J = 6.8 Hz, 3H).

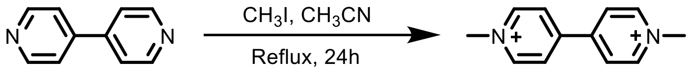

MV^{2+}

Supplementary Figure 5. Synthesis of 1,1'-dimethyl-[4,4'-bipyridine]-1,1'-diium (MV^{2+}).

The synthesis process of MV^{2+} is similar to the preparation process of C_{12}-MV^{2+}.
MV^{2+}: Purple solid, ^{1}H NMR (400 MHz, D_2O) δ 8.96 (d, J = 7.0 Hz, 1H), 8.45 (d, J = 7.0 Hz, 1H), 4.41 (s, 1H). ^{13}C NMR (101 MHz, D_2O) δ 149.86, 146.42, 126.92, 48.68.

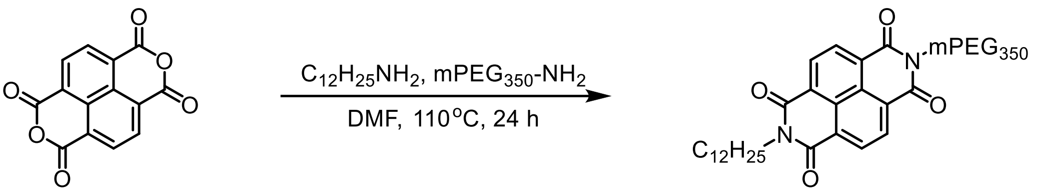

Supplementary Figure 6. Synthesis of 2-dodecyl-7-(2,5,8,11,14,17,20,23-octaoxapentacosan-25-yl)benzo[lmn][3,8]phenanthroline-1,3,6,8(2H,7H)-tetraone (C_{12}-NDI-PEG350).

mPEG350-NH_2 was prepared according to literature[1].

In a 150 mL round-bottom flask, a mixture of 1,4,5,8-naphthalenetetracarboxylic dianhydride (1 g, 3.7 mmol), dodecylamine (0.68 g, 3.7 mmol), and mPEG350-NH_2 (1.3 g, 3.7 mmol) was dissolved in 30 mL of DMF, followed by stirring at 110°C for 24 h. Subsequently, the solution was evaporated, and the resulting mixture was dissolved in dichloromethane (DCM) and washed with 1 M aqueous hydrochloric acid. Pure C_{12}-NDI-PEG350 was finally obtained as a yellow solid through column chromatography (0.6 g, 19 % yield). ^{1}H NMR (400 MHz, Chloroform-*d*) δ 8.74 (s, 4H), 4.44 (t, 2H), 4.21 (m, 2H), 3.83 (t, J = 5.8 Hz, 2H), 3.63 (s, 28H), 3.36 (s, 3H), 1.23 (s, 20H), 0.86 (m, 3H). ^{13}C NMR (101 MHz, Chloroform-*d*) δ 162.94, 131.09, 126.69, 71.99, 70.62, 70.12, 67.87, 59.12, 41.10, 39.61, 31.99, 29.60, 28.16, 27.17, 22.77, 14.22.

3. Preparation of buffer solutions:

Boric acid-potassium chloride-sodium hydroxide buffer solution (pH 8) was prepared by mixing 25 mL of boric acid-potassium chloride (0.2 M) with 4 mL of 0.1 M aqueous sodium hydroxide and then diluting the mixture to 100 mL with water.
Ammonium chloride-ammonia buffer solution (pH 9.18) was prepared by mixing 0.1 mol/L ammonium chloride with 0.1 mol/L ammonia in a 2:1 ratio.
Boric acid-potassium chloride-sodium hydroxide buffer solution (pH 10) was prepared by mixing 25 mL of boric acid-potassium chloride with 43.9 mL of 0.1 M aqueous sodium hydroxide and then dilute the mixture to 100 mL with water.
Disodium hydrogen phosphate-sodium hydroxide buffer solution (pH 12) was prepared by mixing 50 mL of 0.05 M disodium hydrogen phosphate solution with 26.9 mL of 0.1 M aqueous sodium hydroxide solution and then diluting the mixture to 100 mL with water.
Potassium chloride-sodium hydroxide buffer solution (pH 13) was prepared by mixing 25 mL of 0.2 M potassium chloride solution with 66 mL of 0.2 M aqueous sodium hydroxide solution and then diluting the mixture to 100 mL with water.
To monitor the change in solution pH during the experiment, we used a pH meter to measure the pH of buffer solution (pH 12) containing C_{12}-MV^{2+} (3 mM) and PN (3 mM) in the presence of N_2H_4•H_2O (5% v/v) over time. The results indicated that the pH of the buffer solution remained constant throughout the experiment.

4. The influence of viologen derivatives on supramolecular self-assembly.

Firstly, we investigated the influence of viologen derivatives on supramolecular self-assembly. The results showed that when amphiphilic molecules with long hydrophobic polar chains (C_{12}-MV^{2+} or C_{16}-MV^{2+}) were dissolved in water containing PN, the self-assembly could be obtained due to charge transfer (CT) interaction and amphipathic interaction. Inhere, C_{12}-MV^{2+} with better water solubility was used for further studies.

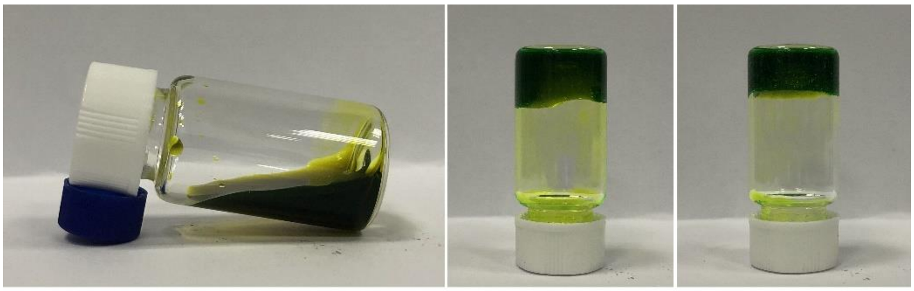

C_8-MV^{2+}/PN **C_{12}-MV^{2+}/PN** **C_{16}-MV^{2+}/PN**

Supplementary Figure 7. Images of viologen derivatives (10 mM) and PN (10 mM) in the aqueous solutions.

5. The influence of pH on supramolecular self-assembly.

The influence of pH on supramolecular self-assembly was investigated by viscosity measurements. The results indicated that when the pH of the solutions was 12, the solutions containing C_{12}-MV^{2+} (10 mM) and PN (10 mM) exhibited the highest viscosity. Therefore, all subsequent measurements were carried out in the buffer (pH = 12).

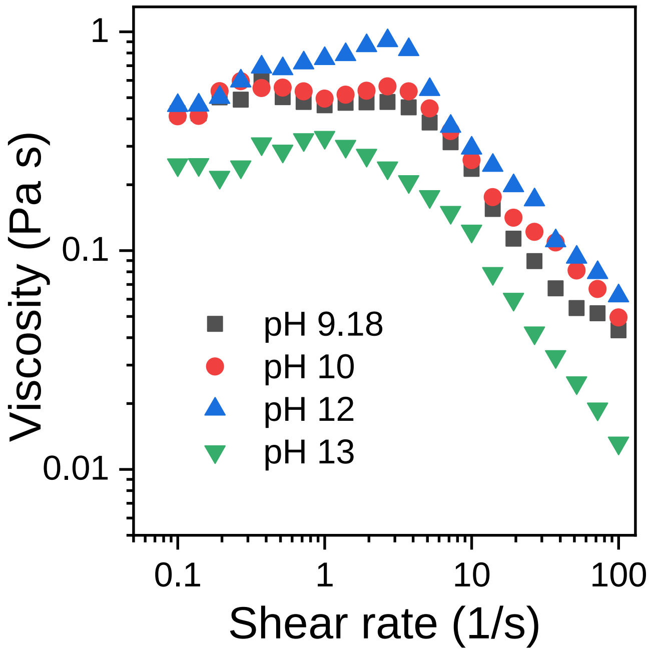

Supplementary Figure 8. Viscosity changes by varying the pH of solutions (10 mM C_{12}-MV^{2+}, 10 mM PN).

6. Supramolecular polymerization via charge transfer and amphiphilic interaction.

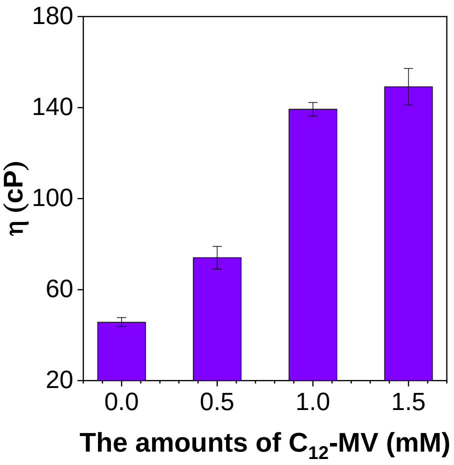

Supplementary Figure 9. Viscosity changes depicting the conformational switching by varying the equivalents of (0 *equiv*, 0.5 *equiv*, 1 *equiv*, and 1.5 *equiv* of C_{12}-MV^{2+} against 1 mM PN). Data are presented as the average values ± s.d. (n=3).

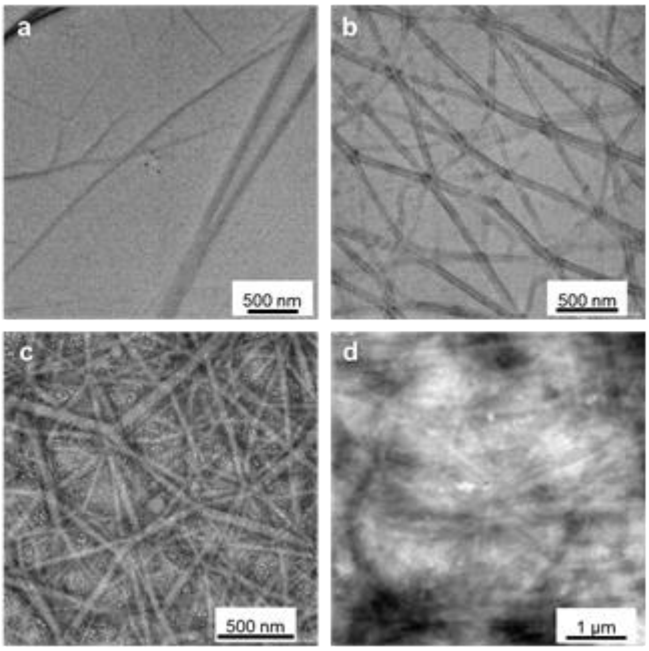

Supplementary Figure 10. TEM images of molar equivalent C_{12}-MV^{2+}/PN at different concentrations. (a) 0.5 mM; (b) 1 mM; (c) 3 mM; (d) 5 mM.

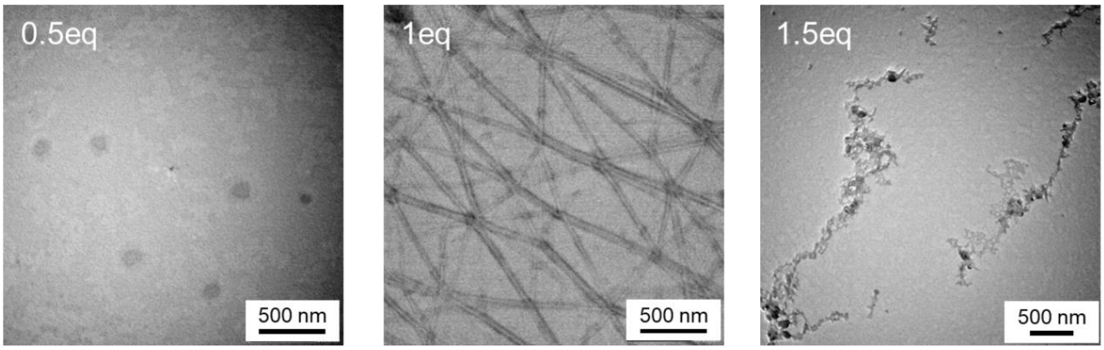

Supplementary Figure 11. Respective TEM images show the transformation from micelles to fiber collected at 0.5 *equiv*, 1 *equiv*, and 1.5 *equiv* of PN against C_{12}-MV^{2+} (1 mM).

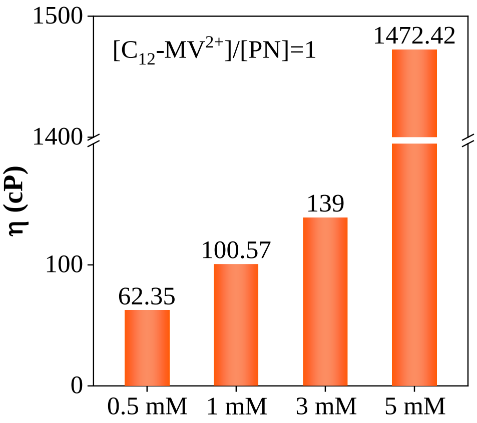

Supplementary Figure 12. Viscosity of the solution at different concentrations of monomer.

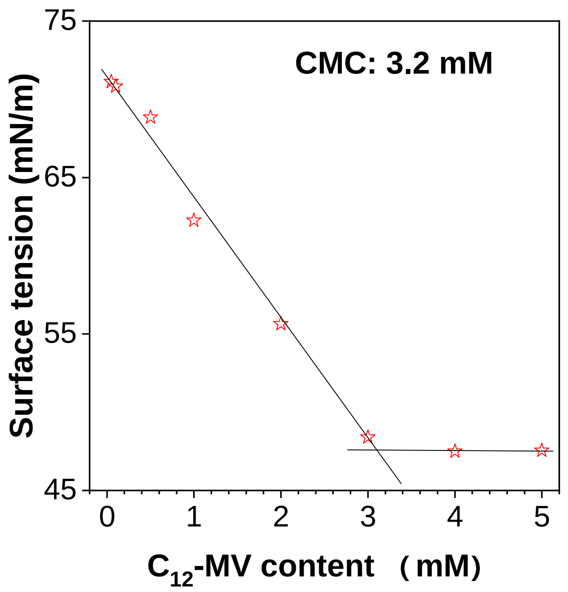

Supplementary Figure 13. The surface tension of different C_{12}-MV^{2+} content in water.

To support the maximum degree of aggregation of the assembly of equimolar C_{12}-MV^{2+}/ PN samples, dynamic light scattering (DLs) was employed to determine the size of assembly. As shown in the Supplementary Figure 14, the results indicate that the assembly reaches its maximum diameter (108 nm) only when the ratio of incoming PN to C_{12}-MV^{2+} is 1.

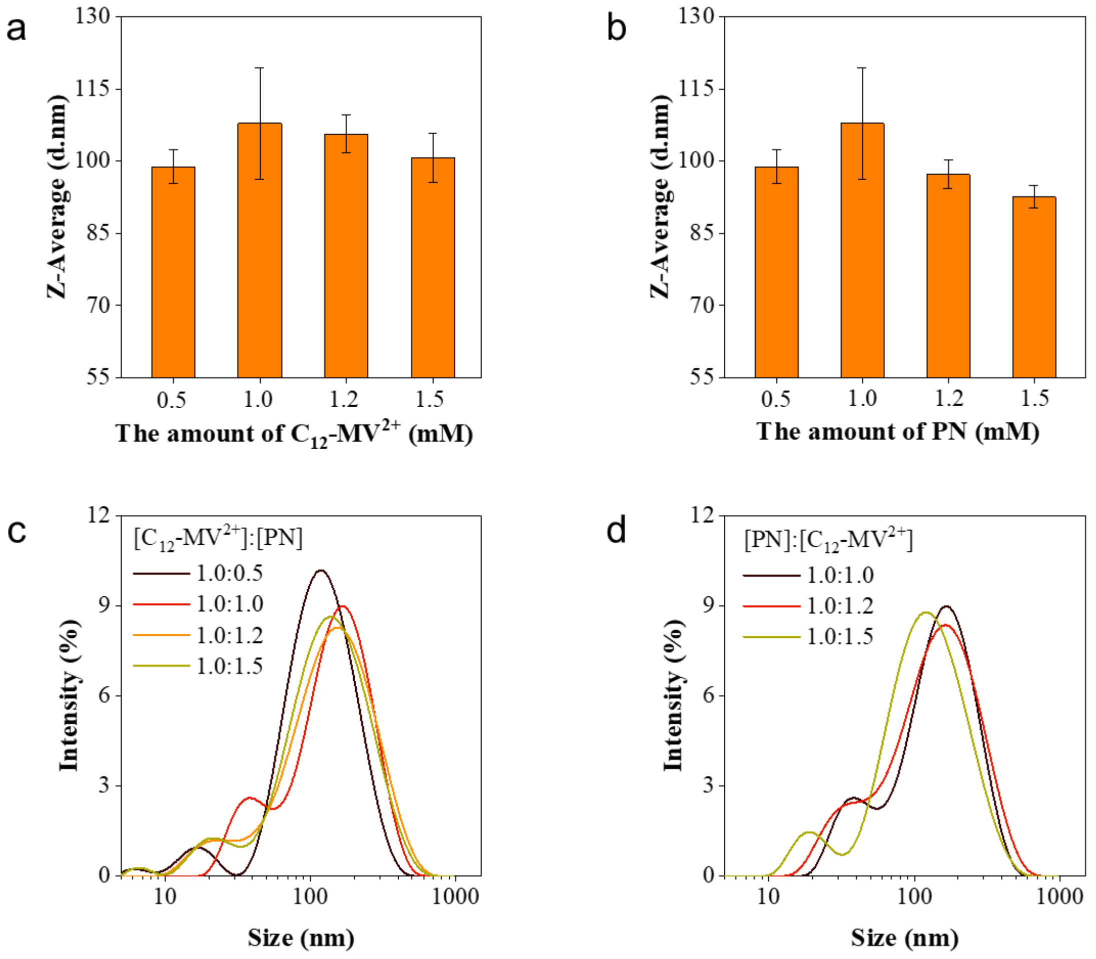

Supplementary Figure 14. Z-average diameter of C_{12}-MV^{2+}-PN aggregates in buffer solution (pH = 12), (a) [PN] = 1 mM, (b) [C_{12}-MV^{2+}] = 1 mM. Data are presented as the average values ± s.d. (n=3). DLs measurements of C_{12}-MV^{2+}-PN aggregates in buffer solution (pH = 12), (c) [PN] = 1 mM, (d) [C_{12}-MV^{2+}] = 1 mM.

7. Redox kinetics of C_{12}-MV^{2+} investigated by UV-vis measurements.

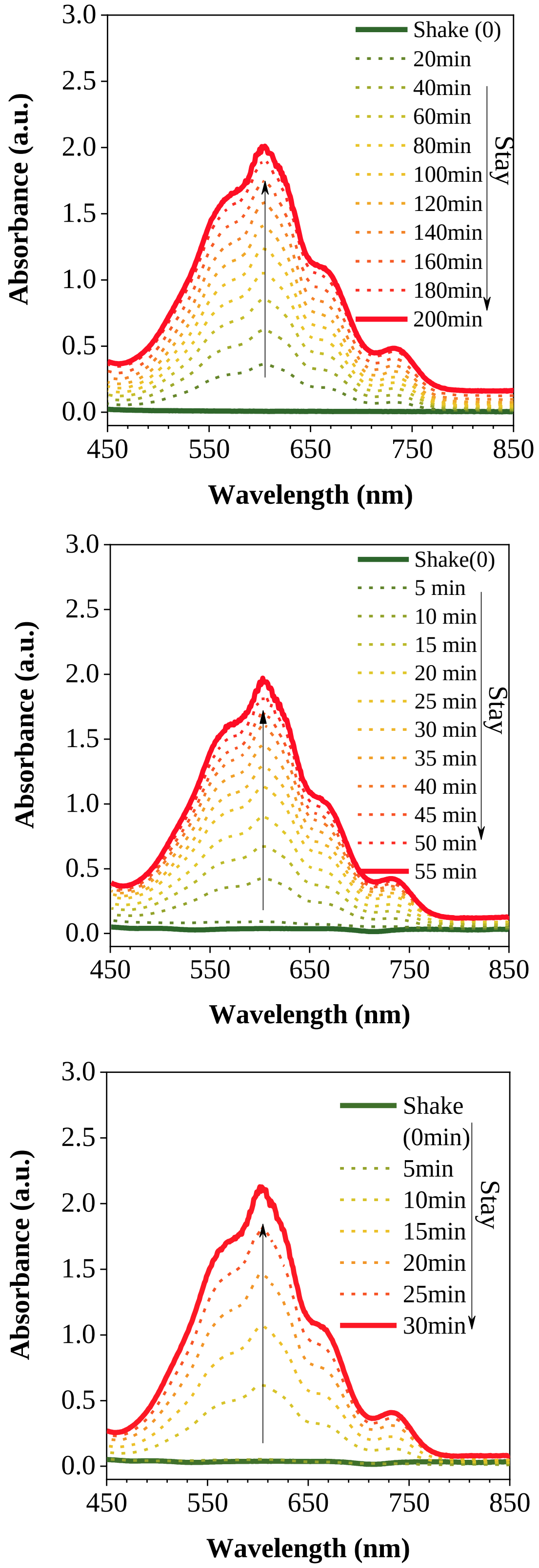

Supplementary Figure 15. Time-dependent UV-vis measurements showing the transient existence of C_{12}-$MV^{\bullet+}$, [C_{12}-MV^{2+}] = 3 mM, with different content of $N_2H_4 \bullet H_2O$ (**5% v/v, 10% v/v, 20% v/v**).

8. Redox kinetics of C_{12}-MV^{2+} investigated by ^{1}H-NMR spectra.

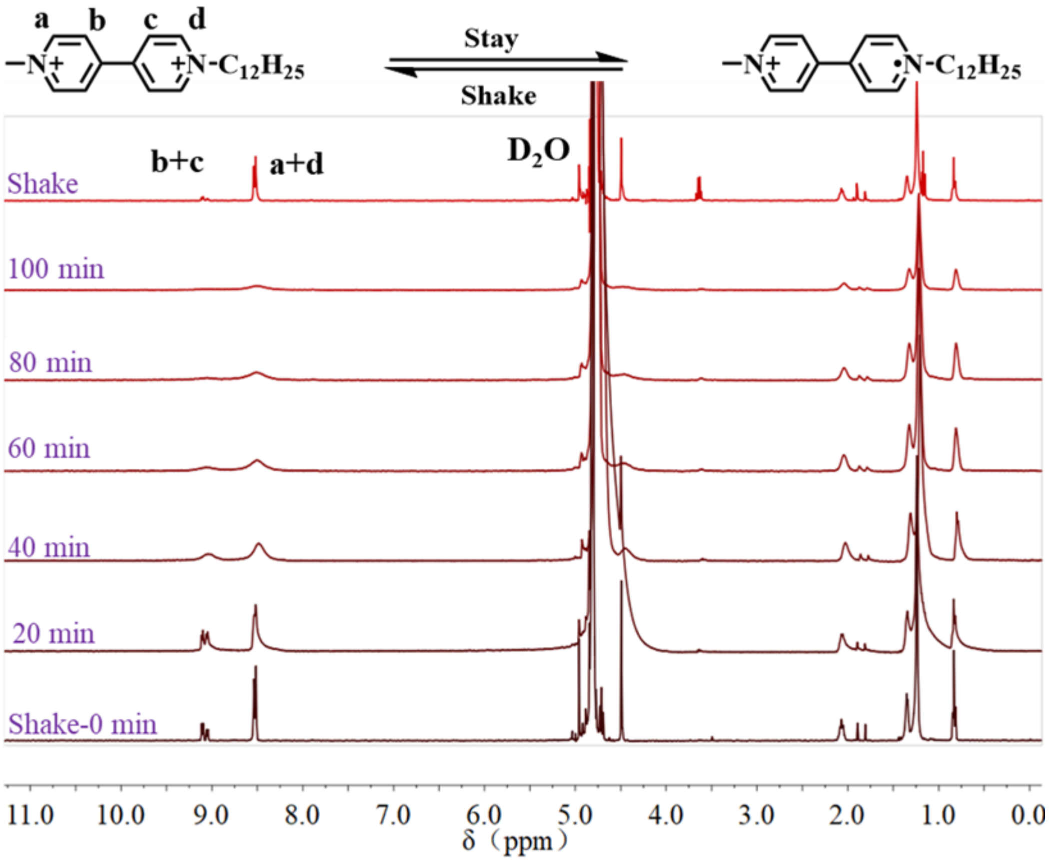

Supplementary Figure 16. Time-dependent ^{1}H-NMR spectra of C_{12}-MV^{2+} and N_2H_4•H_2O at 25 °C.

9. Redox kinetics of C_{12}-MV^{2+} investigated by EPR measurements.

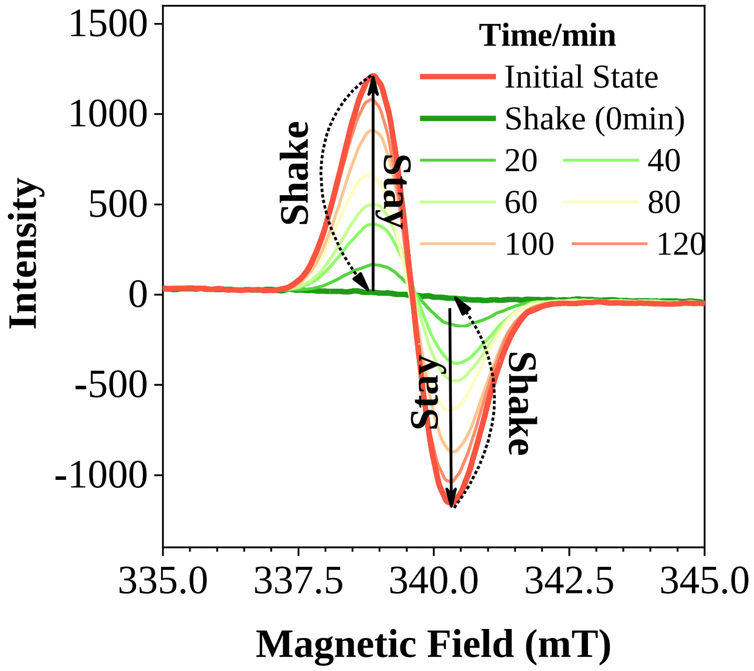

Supplementary Figure 17. Time-dependent EPR measurements showing the transient existence of C_{12}-$MV^{•+}$, [C_{12}-MV^{2+}] = 3 mM.

10. The dissipative performance can be activated again by introducing fresh air in a closed system.

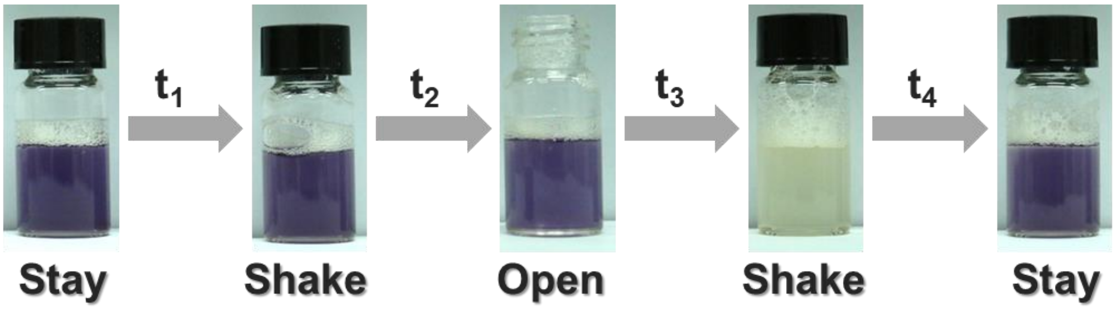

Supplementary Figure 18. Visualization of discoloration activated again by introducing fresh air in a closed system.

11. Gas phase changes in redox processes.

Firstly, we used gas chromatography to monitor the gas composition during the redox process, and the chromatogram showed a gradual decrease in oxygen and a progressive increase in nitrogen content over time (Supplementary Figure 19b). To better track the kinetic changes in gas composition, as shown in Supplementary Figure 19a, we employed an oxygen-nitrogen percentage detector to quantitatively monitor the gas phase change process. The results obtained from the detector were consistent with those obtained from gas chromatography measurements. Additionally, we utilized detectors with ppm-level sensitivity for nitric oxide and nitrogen dioxide to confirmed the absence of nitric oxide and nitrogen dioxide production.

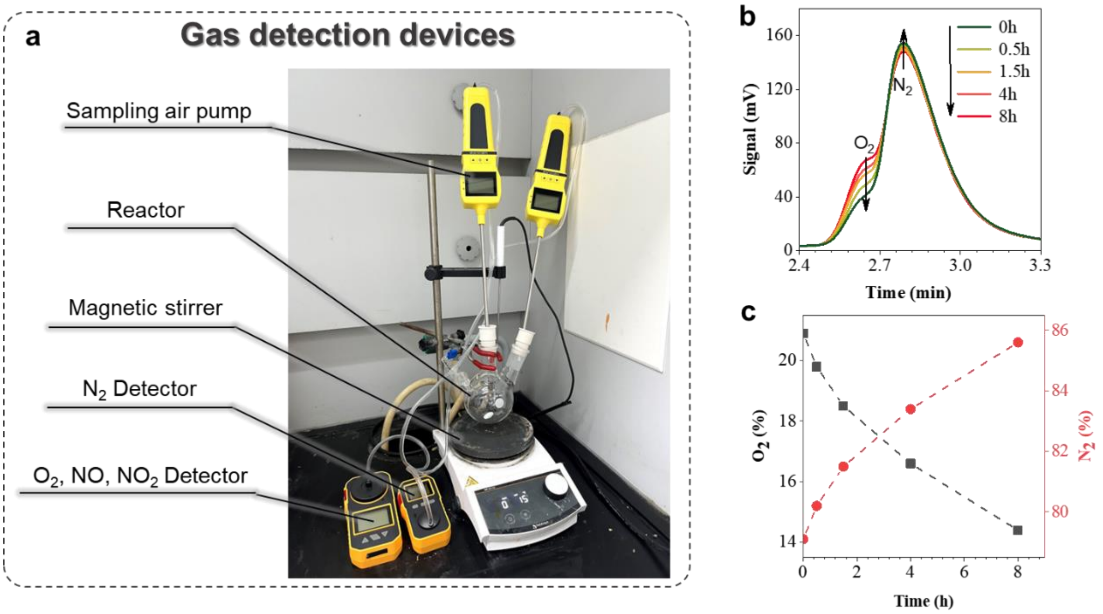

Supplementary Figure 19. (a) The composition of the gas detection equipment. (b) The gas chromatography spectrum displays the change process of oxygen and nitrogen. (c) The gas phase component changes in the redox process were quantitatively obtained through the gas detectors. $[C_{12}\text{-}MV^{2+}]$ = 5 mM, $N_2H_4{\bullet}H_2O$ (10% v/v), V_g:V_l = 3:1.

12. C_{12}-MV^{2+} / PN dissipation states at different times.

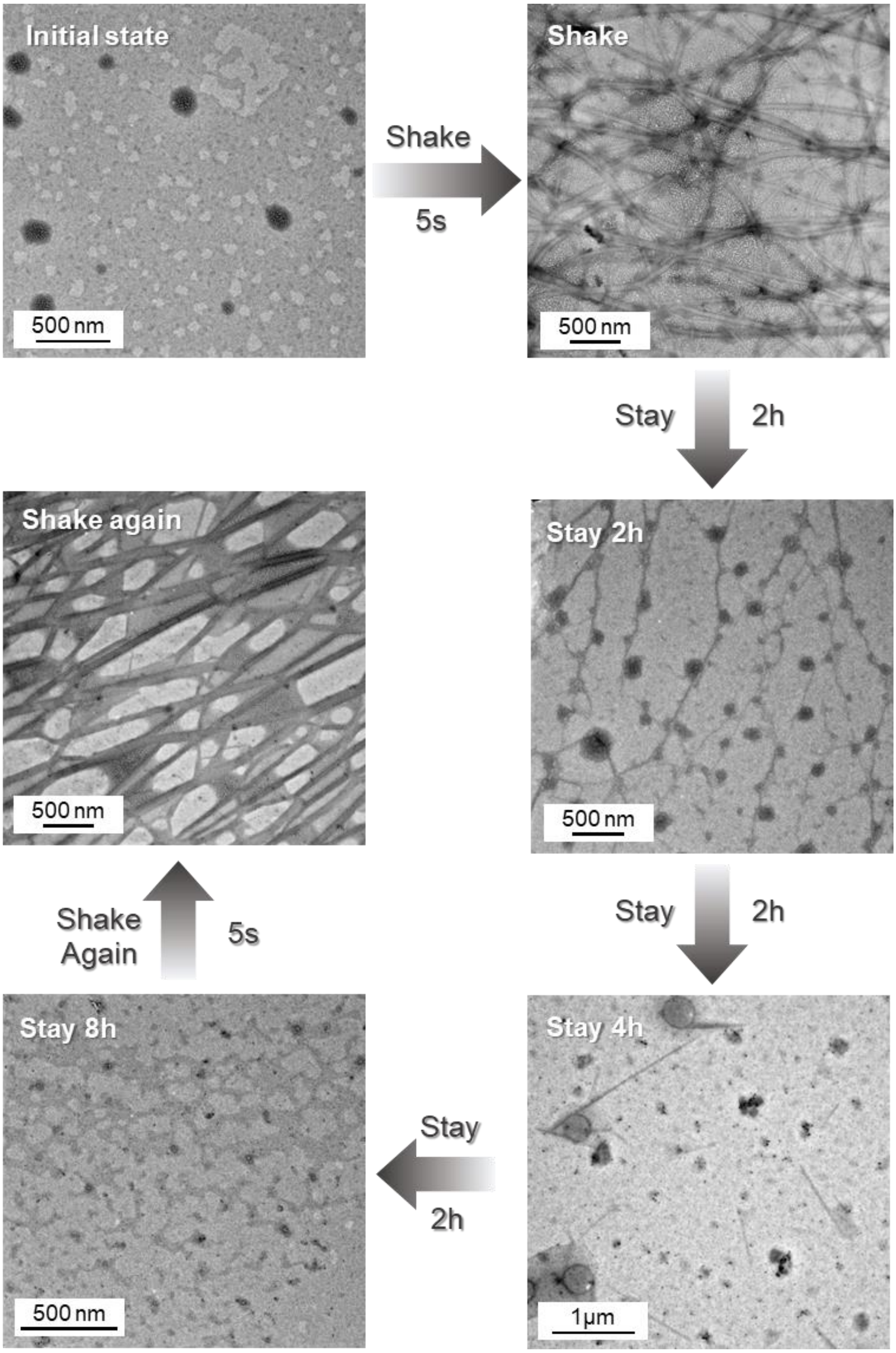

Supplementary Figure 20. C_{12}-MV^{2+}(1mM) / PN(1mM) dissipation states at different times.

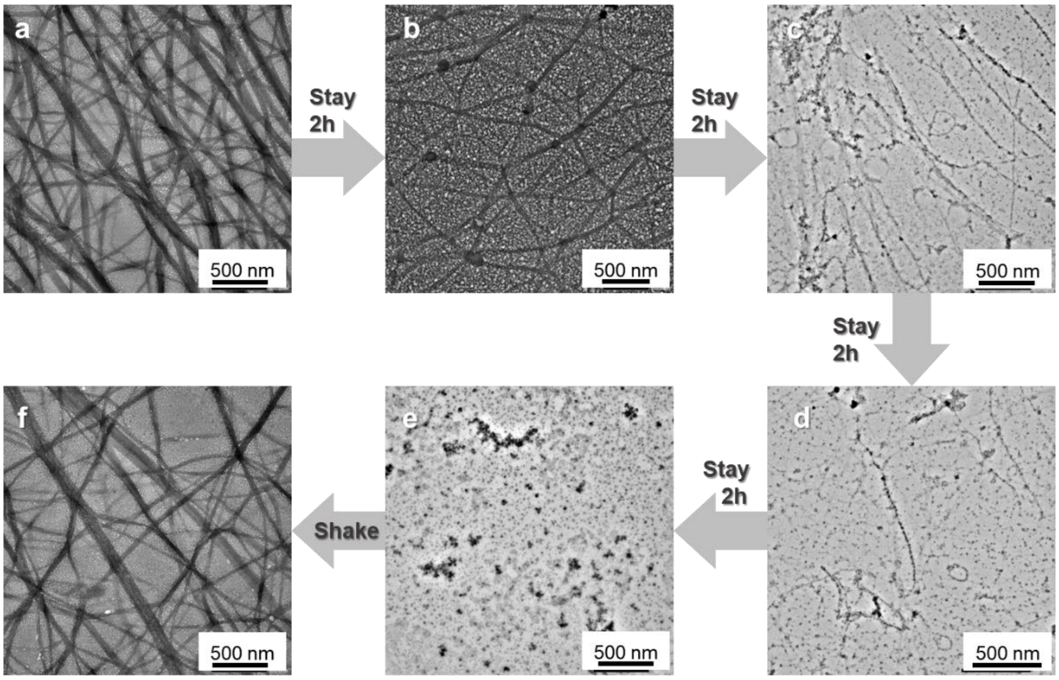

Supplementary Figure 21. (a-f) TEM images of solution with C_{12}-MV^{2+}/ PN (3 mM/ 3 mM) the presence of N_2H_4•H_2O (10% v/v) before and after shake over time.

13. Dissipation states of C_{12}-MV^{2+}/PN induced by phenyl lactate chirality at different times.

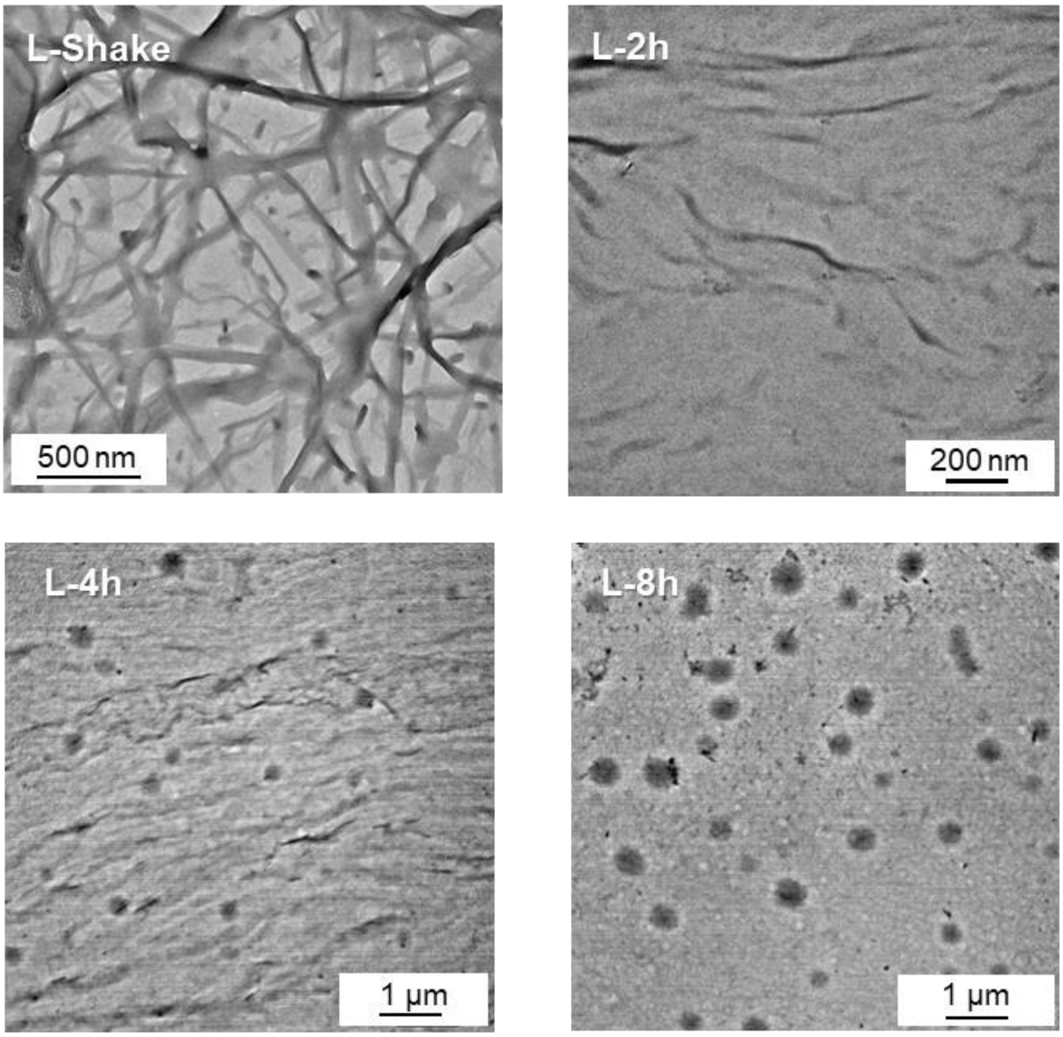

Supplementary Figure 22. Dissipation states of C_{12}-MV^{2+}/PN induced by phenyl lactate chirality at different times**.**

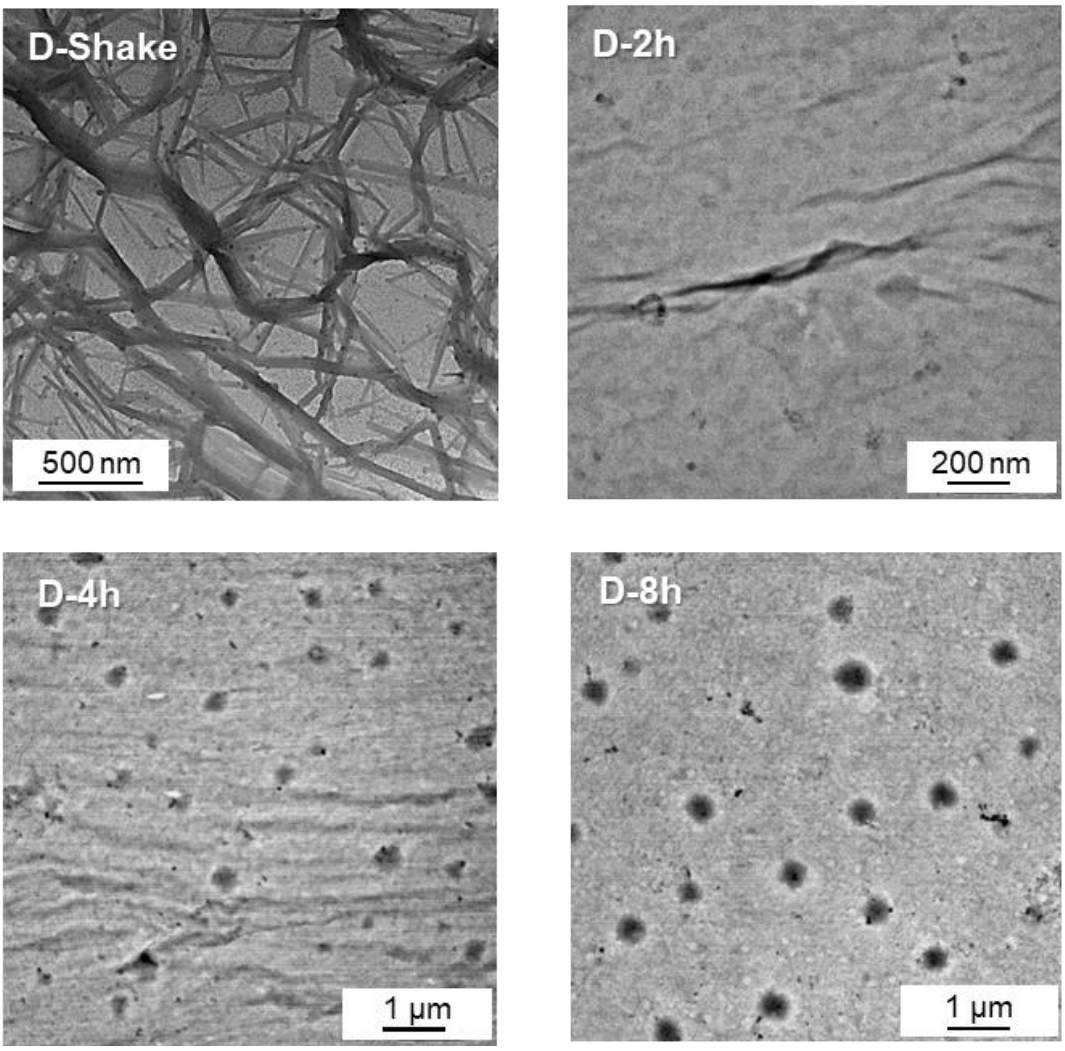

Supplementary Figure 23. Dissipation states of C_{12}-MV^{2+}/PN induced by phenyl lactate chirality at different times.

14. Redox kinetics of C_{12}-MV^{2+}/PN investigated by EPR measurements.

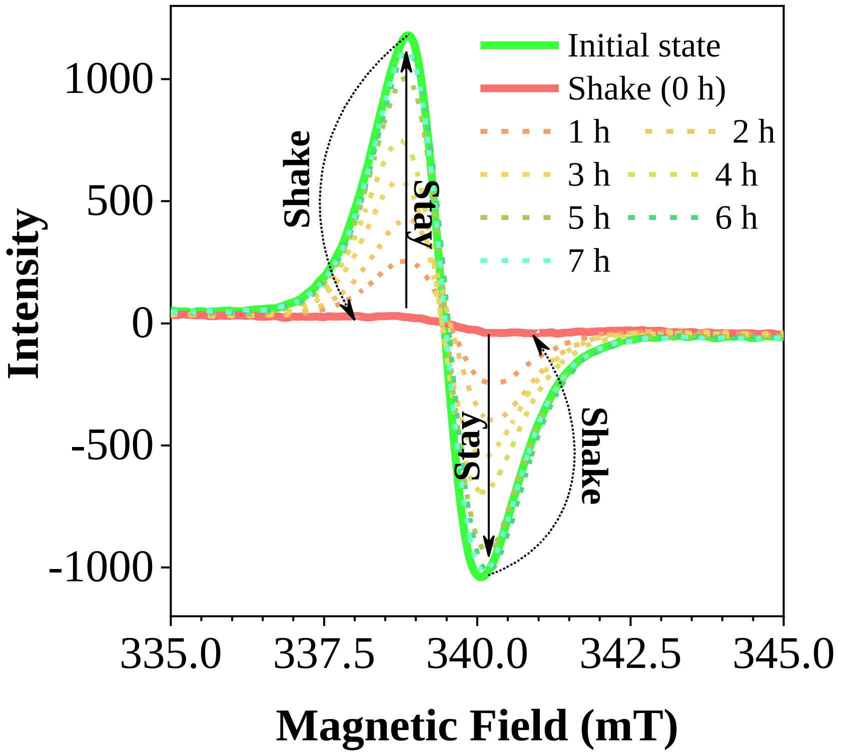

Supplementary Figure 24. Time-dependent EPR measurements show the existence of C_{12}-$MV^{\bullet+}$, [C_{12}-MV^{2+}] = 1 mM, [PN] = 1 mM.

15. Redox kinetics of C_{12}-MV^{2+}/PN in the presence of chiral molecules investigated by UV-vis measurements.

We investigated the time-dependent UV-vis spectra of shake-driven transient supramolecular helical structure in the presence of L- phenyllactic acid and D-phenyllactic acid, respectively. As shown in Supplementary Figure 24, we did not observe a significant red shift or blue shift in the spectra. We believed that a red or blue shift in the CD spectra was random, which might be the result of the unstable self-assembled structures under the non-equilibrium state.

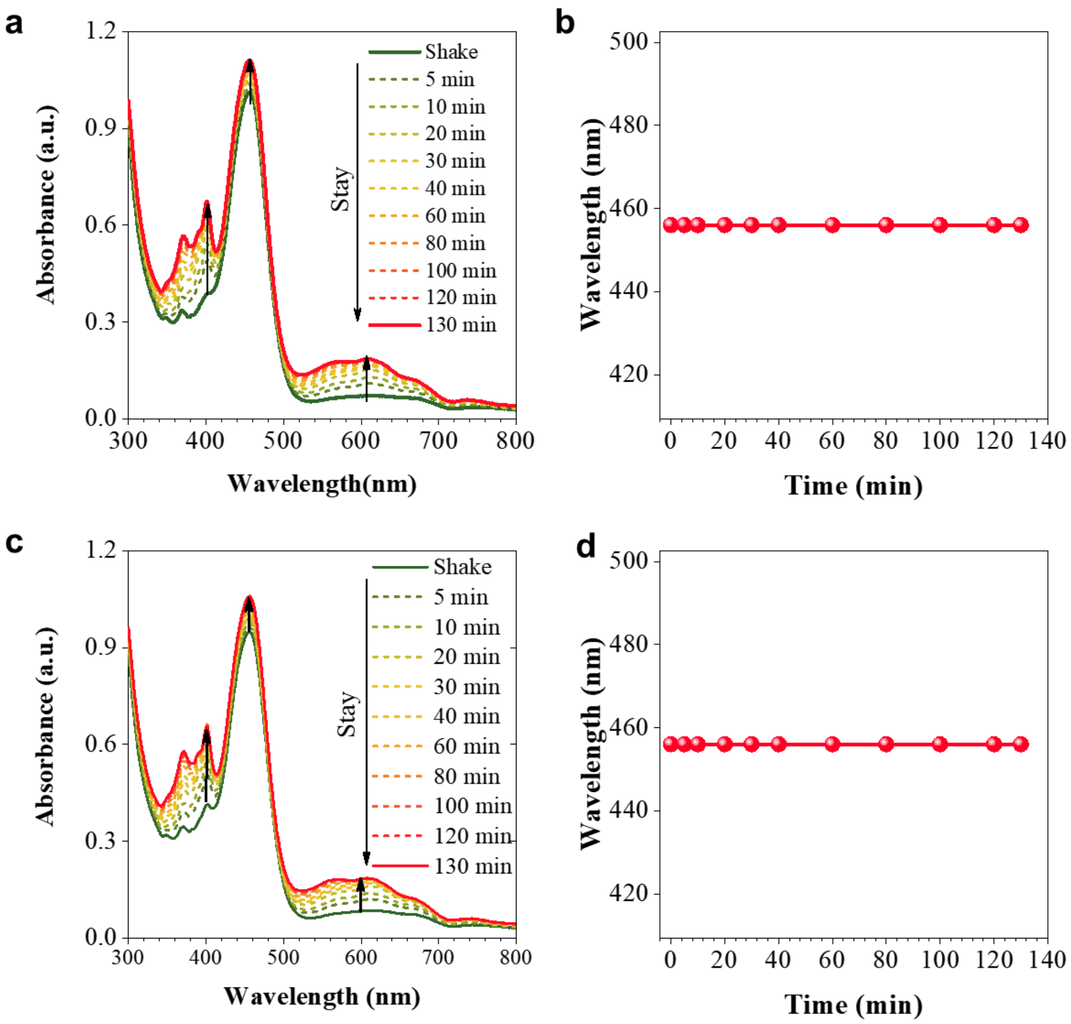

Supplementary Figure 25. Time-dependent changes in UV-vis spectra demonstrating shake-driven a temporal helical polymer in the presence of (a) *L*-(-)-Phenyllactic acid or (c) *D*-(+)-Phenyllactic acid (N_2H_4•H_2O, 5% v/v). (b) The maximum absorbance wavelength change of shake driven transient polymer over time in the presence of (b) *L*-(-)-Phenyllactic acid or (d) *D*-(+)-Phenyllactic acid.

16. Fluorescence quantum yields

$$\phi_f = \frac{N_{em}(\lambda_{ex})}{N_{abs}(\lambda_{ex})}$$

The fluorescence quantum yield, ϕ_f, is the ratio of the number of emitted photons $N_{em}(\lambda_{ex})$to the number of absorbed photons $N_{abs}(\lambda_{ex})$.

Here the fluorescence quantum yield of the solution system before and after C_{12}-MV redox was calculated by comparing the absorption peak area of PN in the UV spectrum and the emission peak area in the fluorescence spectrum with the fluorescence quantum yield of PN itself 0.56[2].

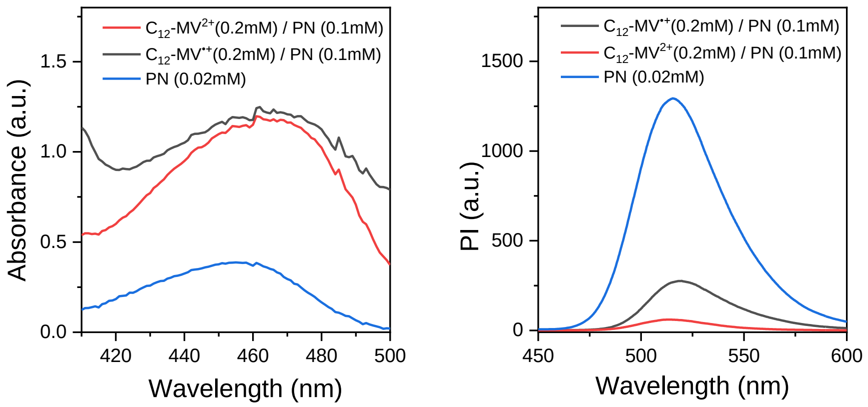

Supplementary Figure 26. UV absorption spectra of the C_{12}-MV^{2+}/PN system before and after oxidation and PN (left). Fluorescence spectra of the C_{12}-MV^{2+}/PN system before and after oxidation and PN (right).

Supplementary Table 1. Calculation of fluorescence quantum yields

	S_{FL}	S_{UV}	S_{FL} / S_{UV}	φ_f of PN	Coefficient	φ_f
PN	70264.3	7.4	9498.29	0.56	5.90E-05	-
C_{12}-$MV^{+•}$/PN	8663.5	35.8	242.00	-	-	0.0143
C_{12}-MV^{2+}/PN	1588.4	32.1	49.48	-	-	0.0029

S_{FL} : Fluorescence peak area; S_{UV} :UV peak area of PN.

17. Fluorescence of PN could be quenched by the CT interaction with C_{12}-MV^{2+}.

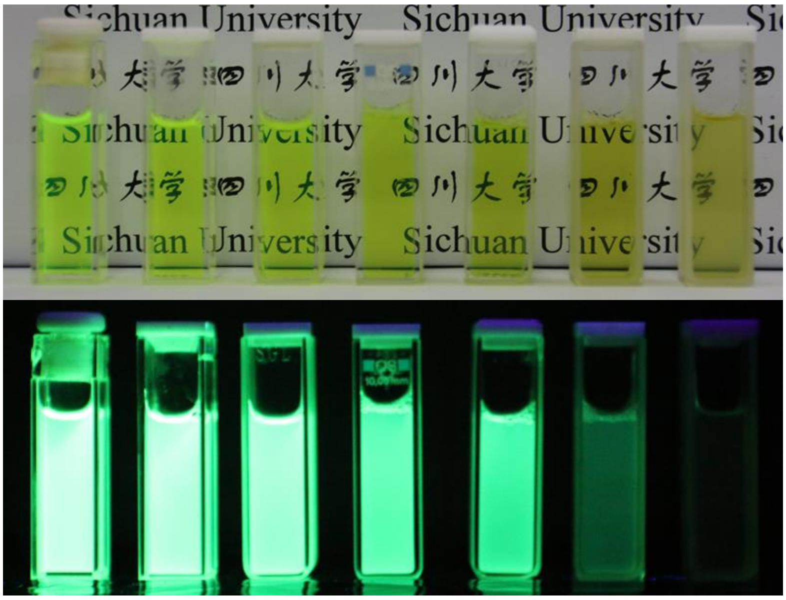

Supplementary Figure 27. Images (upon) and fluorescence images (down) of a solution of PN (0.1 mM) upon addition of increasing concentrations of C_{12}-MV^{2+} aqueous solution (0–5 eq). [C_{12}-MV^{2+}] = 3 mM, pH = 12.

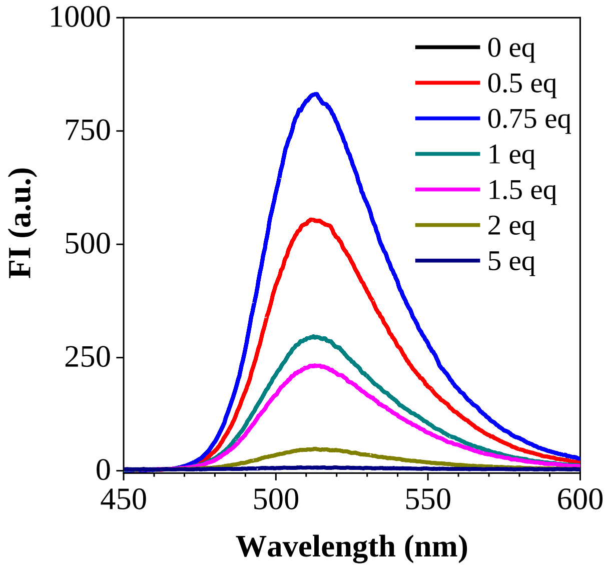

Supplementary Figure 28. Fluorescence spectra of a solution of PN (0.1 mM) upon addition of increasing concentrations of C_{12}-MV^{2+} aqueous solution (0–5 eq).

18. Redox kinetics of MV^{2+}/PN investigated by fluorescence spectroscopy.

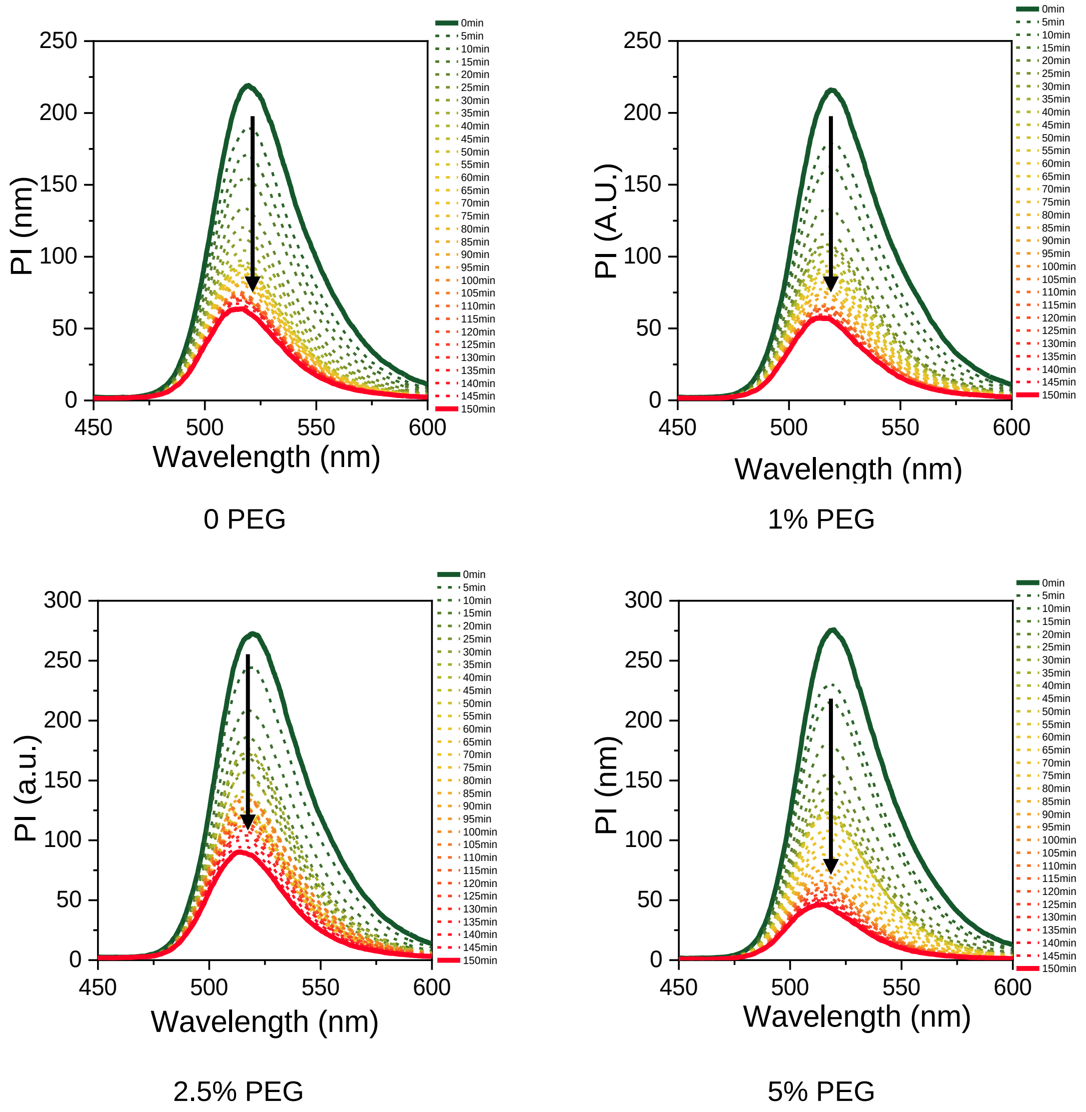

Supplementary Figure 29. Time-dependent fluorescence spectroscopy, $[MV^{2+}]$ = 2 mM, [PN] = 0.2 mM, $N_2H_4 \cdot H_2O$ (20% v/v), with different content of PEG (10kDa).

19. The fluorescence of PN is absorbed by MV^{2+}.

The quenching of PN fluorescence is due to the energy transfer between $MV^{\bullet+}$ and PN. The enhanced fluorescence induced by shake is due to the energy transfer between MV^{2+} and PN was decreased.

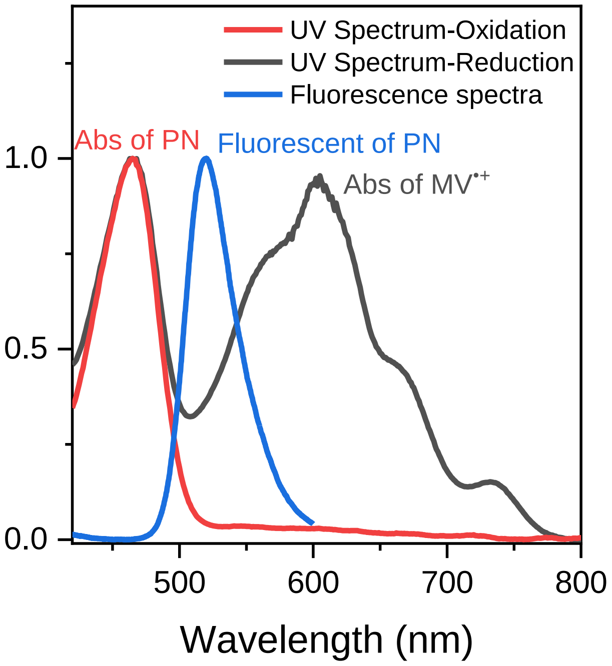

Supplementary Figure 30. UV-vis spectra of MV^{2+} and $MV^{\bullet+}$; Fluorescence spectra of PN.

20. Redox kinetics of MV^{2+} investigated by discoloration.

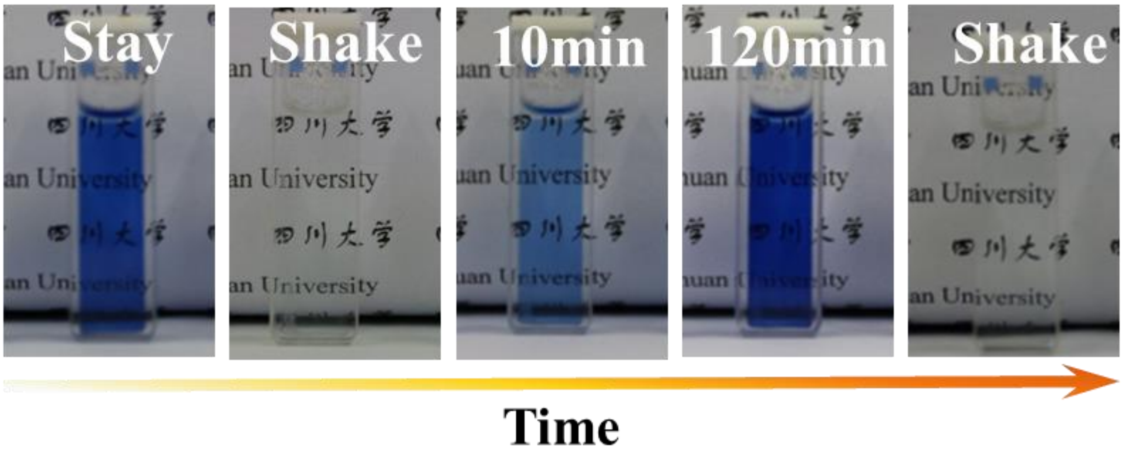

Supplementary Figure 31. Photographs of shake power discoloration over time indicating reversible redox reaction of MV^{2+} by adding N_2H_4•H_2O, $[MV^{2+}]$ = 3 mM, N_2H_4•H_2O (20% v/v), buffer (pH=12).

21. Redox kinetics of MV^{2+} investigated by UV-vis measurements.

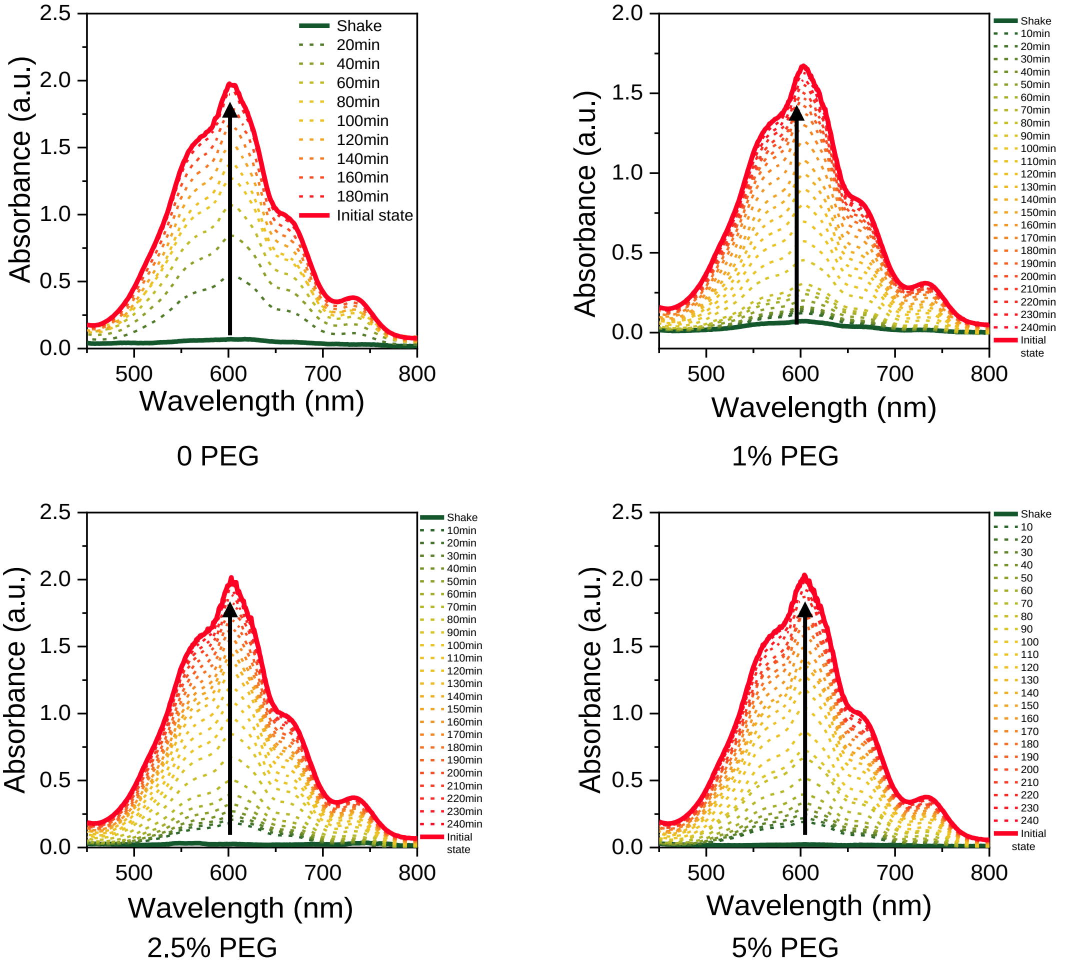

Supplementary Figure 32. Time-dependent UV-vis measurements showing the transient existence of $MV^{\bullet+}$, $[MV^{2+}]$ = 3 mM, $N_2H_4{\bullet}H_2O$ (20% v/v), with different content of PEG (10kDa).

22. Redox kinetics of MV^{2+}/PN investigated by UV-vis measurements.

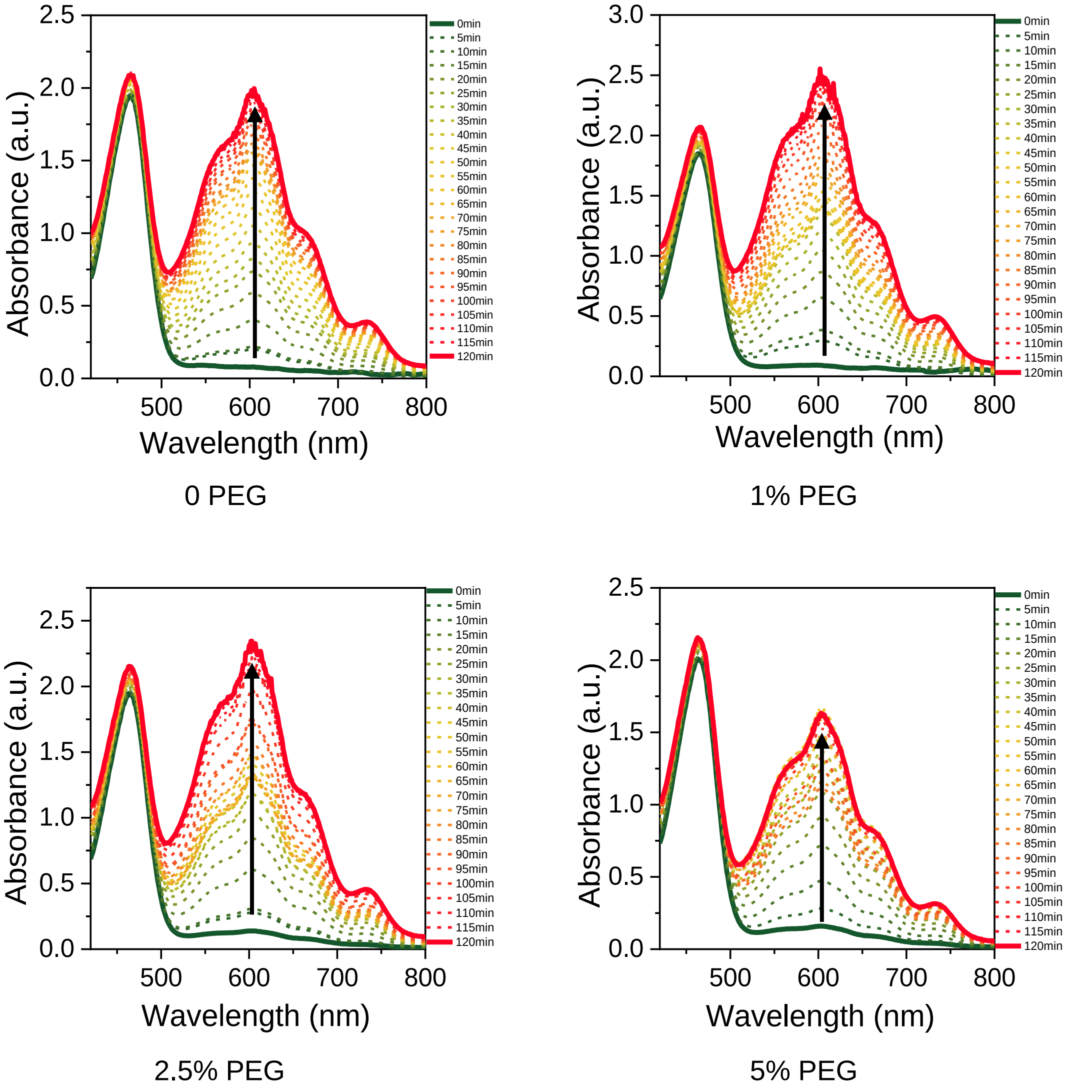

Supplementary Figure 33. Time-dependent UV-vis measurements showing the transient existence of $MV^{\bullet+}$, $[MV^{2+}]$ = 2 mM, [PN] = 0.2 mM, $N_2H_4 \bullet H_2O$ (20% v/v), with different content of PEG (10kDa).

23. Ultrasound-induced patterning.

We observed that oxygen entered the system with a fixed flow direction for directional oxidation during sonication, but after a little staying, instead of showing a flow line of oxygen entry, a pattern of snowflake spots appeared. We consider this because the viscosity of the system is so weak, which makes the flow line discontinuous. Therefore, a series of streamlined patterns were obtained by adding different levels of PEG (10 kDa) into the solution to increase the viscosity of the system. When the PEG concentration reached 5%, a complete heart-shaped streamline pattern was shown.

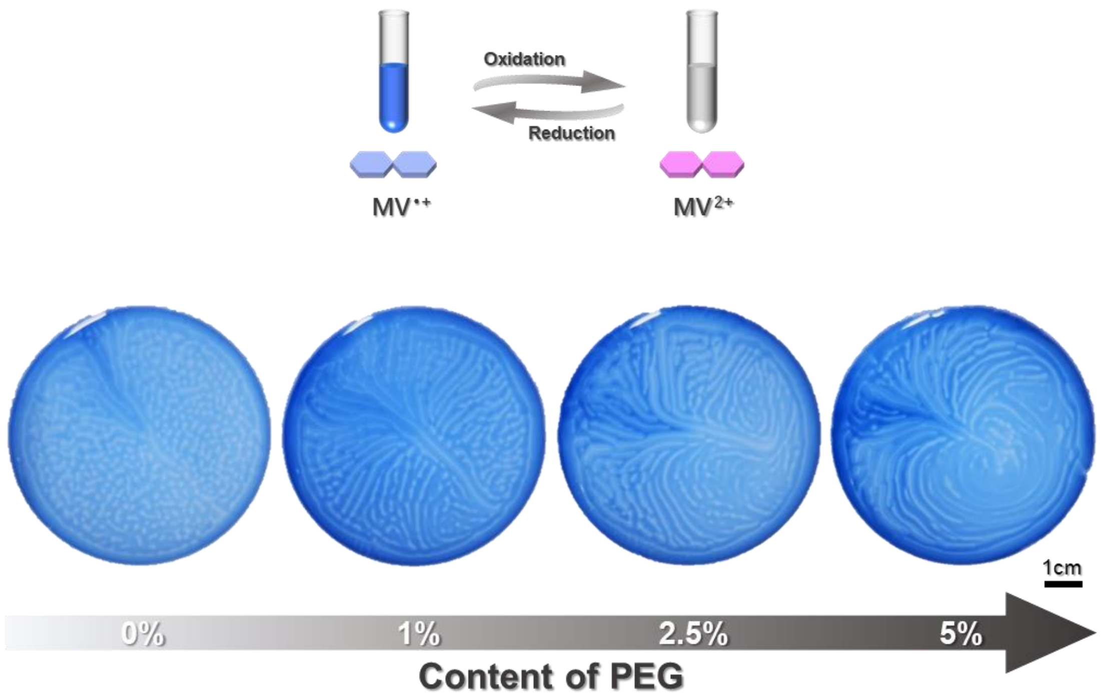

Supplementary Figure 34. Ultrasound-induced patterning of MV^{2+} (5 mM) with different content of PEG (10 kDa). N_2H_4•H_2O (20% v/v). Obtained by sonication for 150 s and stay for 60 s.

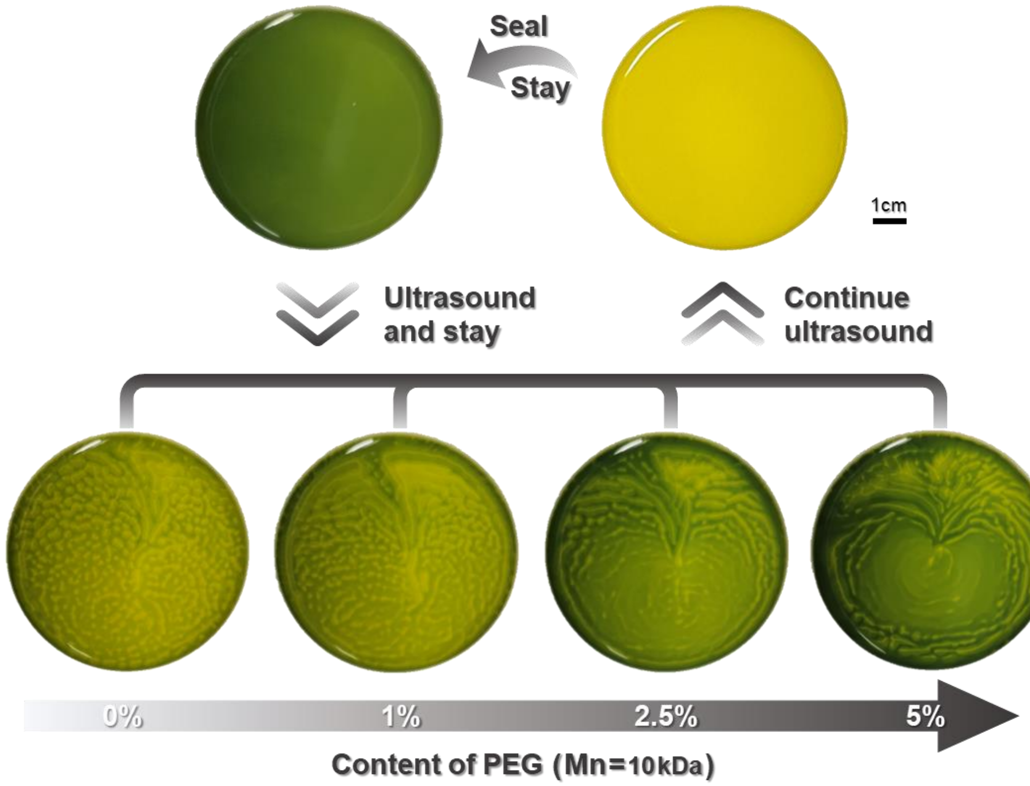

Supplementary Figure 35. Ultrasound-induced patterns for MV^{2+}/PN in buffer containing different contents of PEG, under daylight. Obtained by sonication for 150 s and stay for 60 s.

Compared to MV^{2+}, C_{12}-MV^{2+} itself has surface activity, which increases the viscosity of the system and decreases the surface tension of the system, so the direction of oxygen inflow can be observed without additional PEG addition. Because different concentrations of C_{12}-MV^{2+} have different effects on the surface tension of the system, we observed different sizes of the cardioid flow lines, which decrease with the decrease of surface tension.

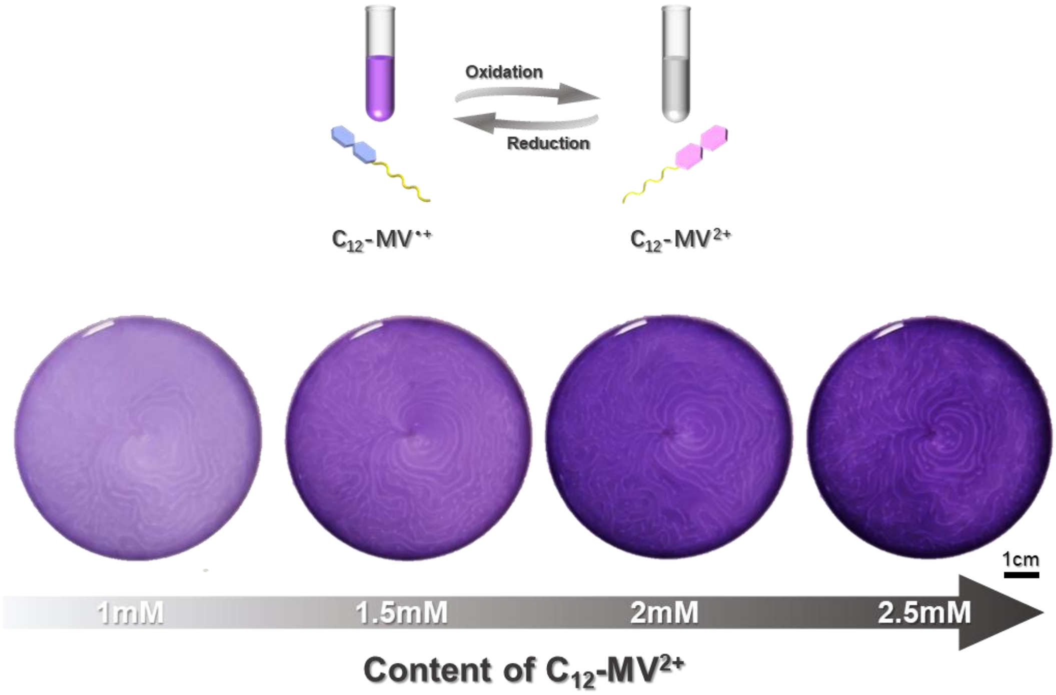

Supplementary Figure 36. Ultrasound-induced patterning of different content of C_{12}-MV^{2+}. N_2H_4•H_2O (20% v/v). Obtained by sonication for 150 s and stay for 60 s.

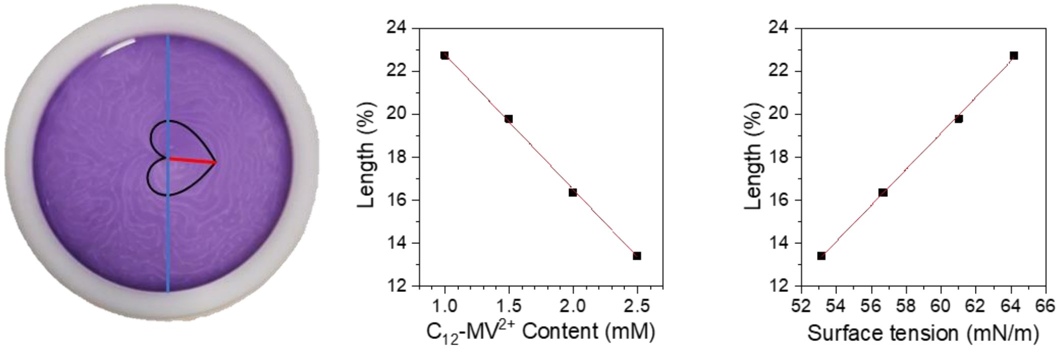

Supplementary Figure 37. Variation of relative size of heart pattern with concentration and surface tension. Relative size is calculated as the ratio of the midline to the diameter.

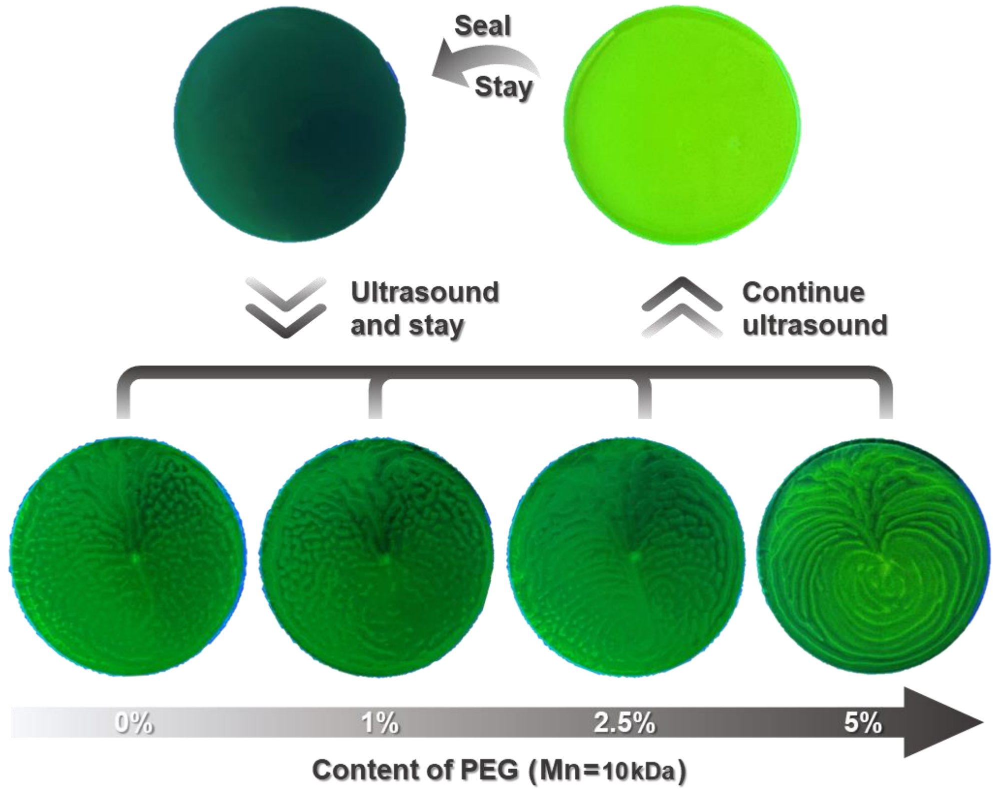

Supplementary Figure 38. Ultrasound-induced patterns for MV^{2+}/PN in buffer containing different contents of PEG, under 365 nm UV light. Obtained by sonication for 150 s and stay for 60 s.

Due to the high viscosity of the C_{12}-MV^{2+}/PN supramolecular system, the force of ultrasound is not sufficient to disperse the reduced C_{12}-$MV^{\bullet+}$ again and it is difficult to form patterns. There is no effective pattern not only in visible light or UV light.

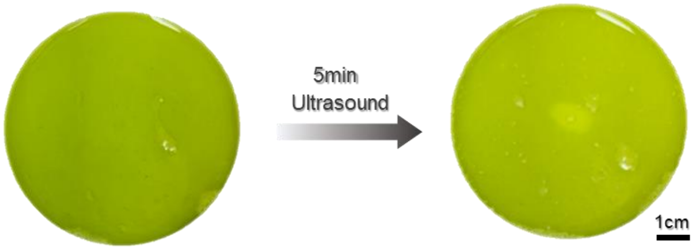

Supplementary Figure 39. Ultrasound-guided patterns for C_{12}-MV^{2+} (1mM) / PN (1mM) in buffer, under daylight. N_2H_4•H_2O (20% v/v). Some of the bright spots are the bubbles.

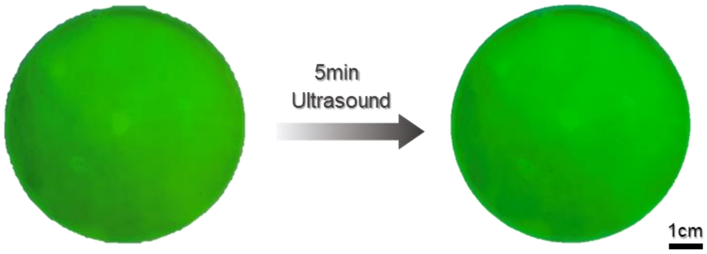

Supplementary Figure 40. Ultrasound-guided patterns for C_{12}-MV^{2+} (1mM) / PN (1mM) in buffer, under 365 nm UV light. N_2H_4•H_2O (20% v/v). Some of the bright spots are the bubbles.

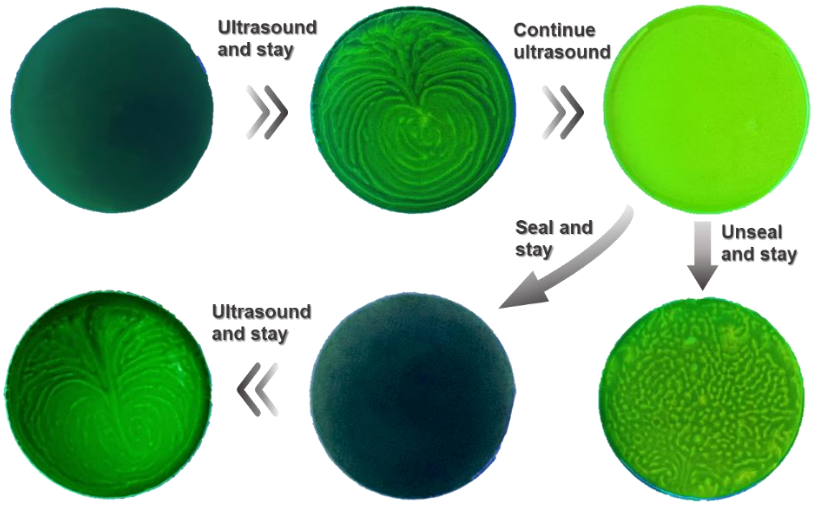

Supplementary Figure 41. Reproducible ultrasound-induced patterns for MV^{2+}/PN in buffer containing 5% contents of PEG, under 365 nm UV light.

24. Reasons for the patterning induced by ultrasound.

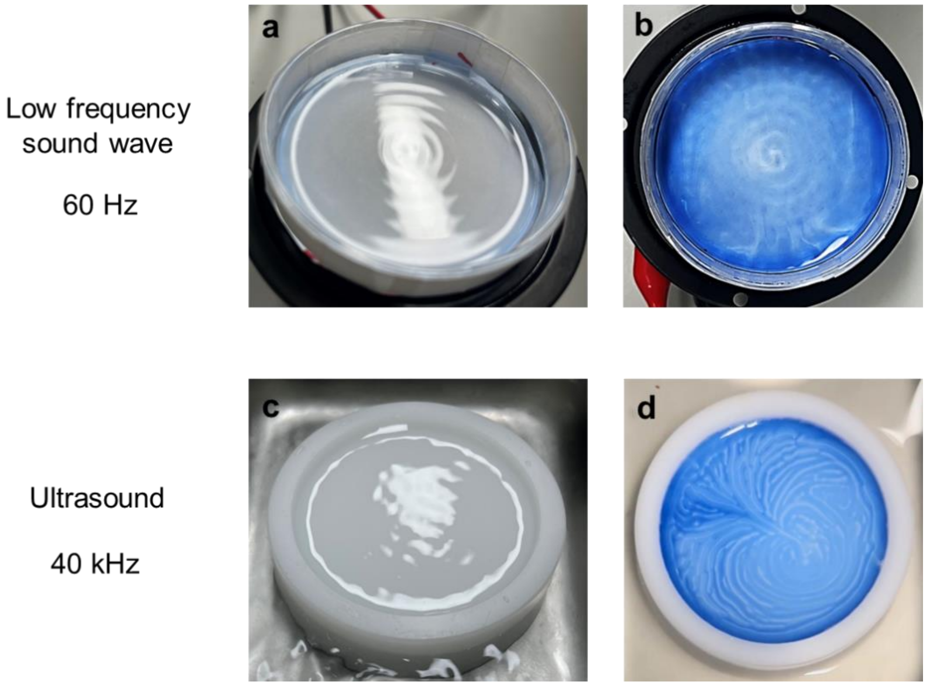

Supplementary Figure 42. (a) Regular vibrational pattern on the surface of the solution under low-frequency vibrations, as displayed by light reflection. (b) Spatiotemporal pattern formed under low-frequency vibrations. (c) Chaotic vibrational pattern on the surface of the solution under ultrasonic vibrations, as displayed by light reflection. (d) Spatiotemporal pattern formed under ultrasonic vibrations.

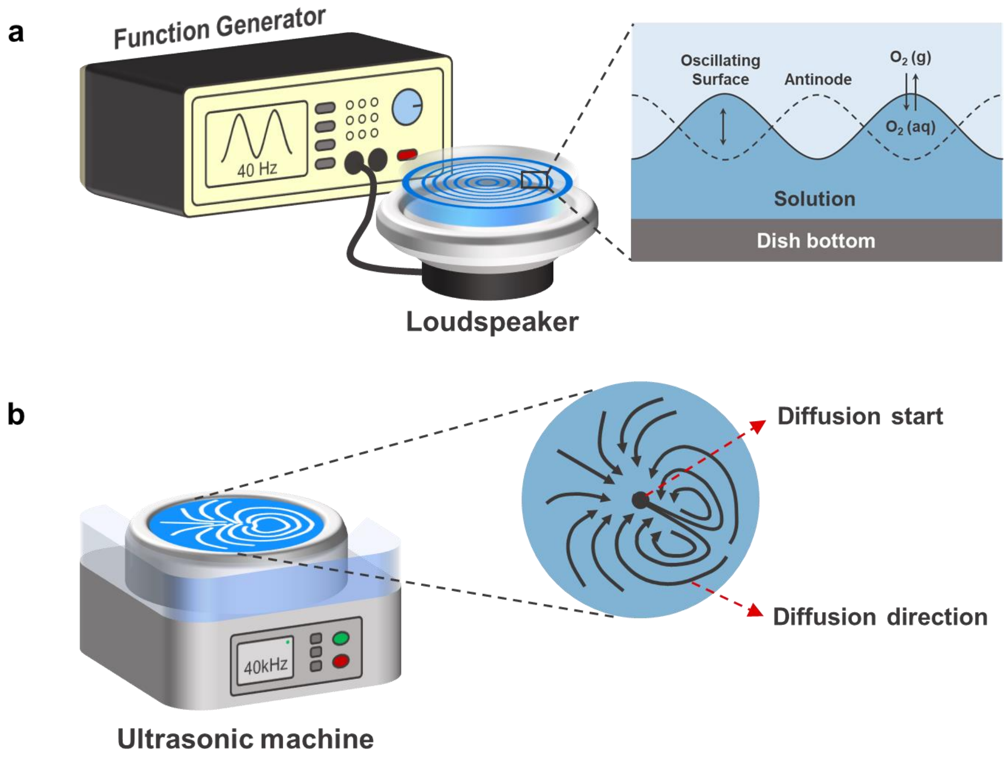

Supplementary Figure 43. Proposed mechanisms of sound-induced patterns. (a) Schematic of the experimental setup for audible sound-controlled pattern generation experiment (left). Schematic cross-sectional view of region-specific dissolution of gases into a vertically vibrating solution (right). (b) Schematic illustration of ultrasound-induced oxygen directed diffusion to form patterns.

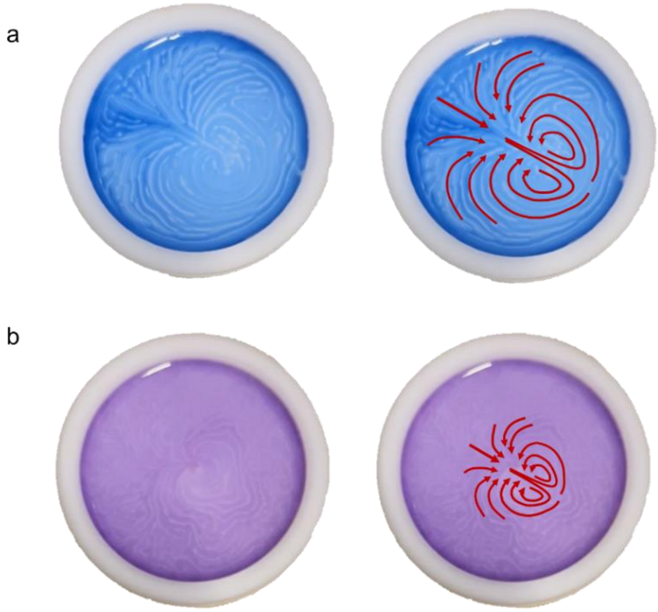

Supplementary Figure 44. The images show the diffusion direction of O_2 during patterning induced by ultrasound. (a) Ultrasound-induced patterning of MV^{2+} (5 mM), PEG (10 kDa, 5%). (b) Ultrasound-induced patterning of C_{12}-MV^{2+} (5 mM). Obtained by sonication for 150 s and stay for 60 s. N_2H_4•H_2O (20% v/v).

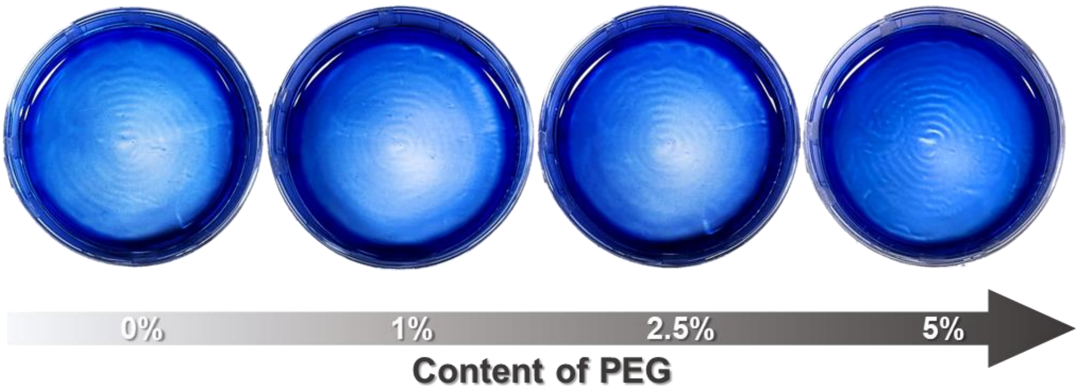

Supplementary Figure 45. Photographs depicting the effect of varying viscosities on pattern formation in the presence of low frequency acoustic vibrations (60Hz, audio-sound). MV^{2+} (5 mM) with different content of PEG (10 kDa), $N_2H_4 \bullet H_2O$ (20% v/v).

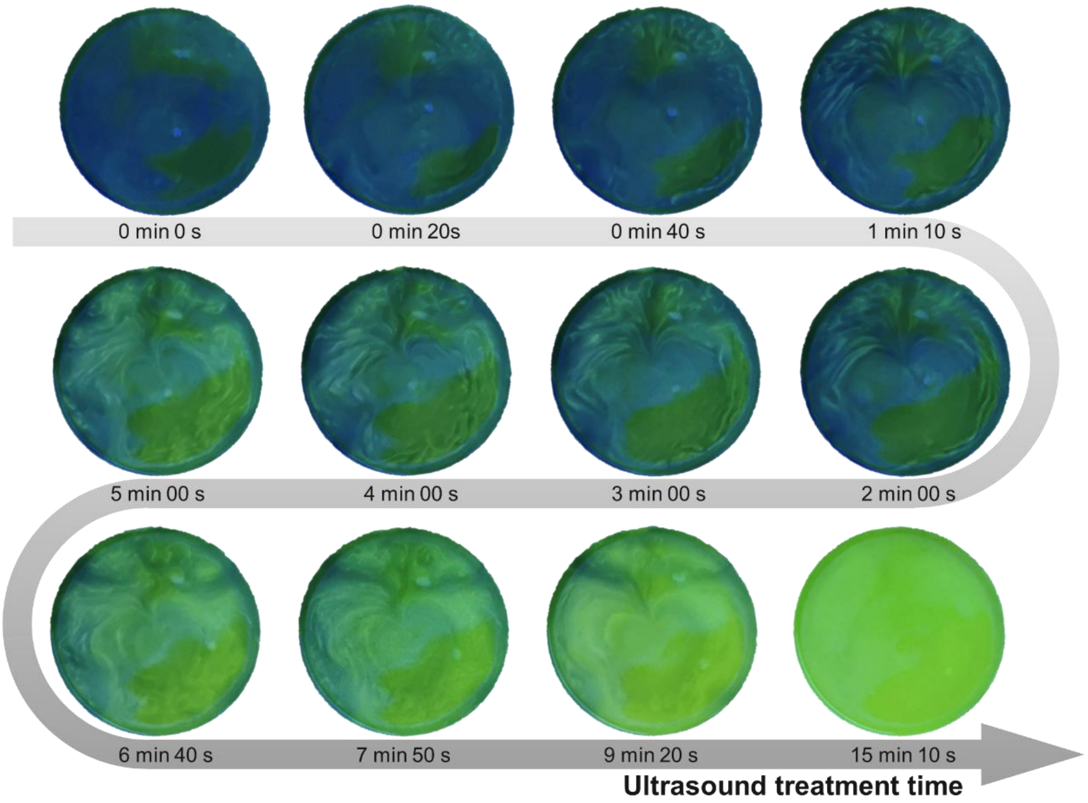

Supplementary Figure 46. Photographs showing fluorescence patterns generation over time induced by continuous ultrasound (40 kHz), under 365 nm UV light. MV^{2+}/PN (5 mM/ 5 mM) in buffer containing 5% PEG (10kDa).

25. Ultrasound-induced patterning in frequencies.

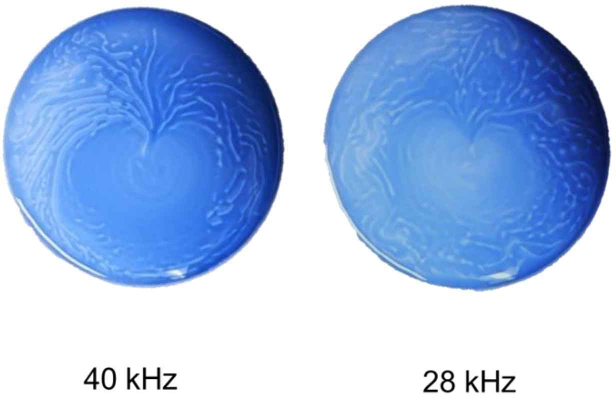

Supplementary Figure 47. Changes in patterns obtained at different ultrasound frequencies. MV^{2+} (5 mM) solution with 2.5% PEG (10 kDa).

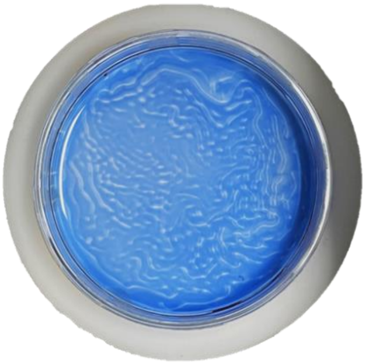

Supplementary Figure 48. Ultrasound destroys the regular pattern formed by low frequency vibrations. MV^{2+} (5 mM) solution with 2.5% PEG (10 kDa).

26. Ultrasound-induced patterning in different molds.

For different shapes of molds (triangular and square), local diffusion processes of ultrasonically induced oxygen entry can be observed, but without an overall large-scale regular patterning relative to circular molds.

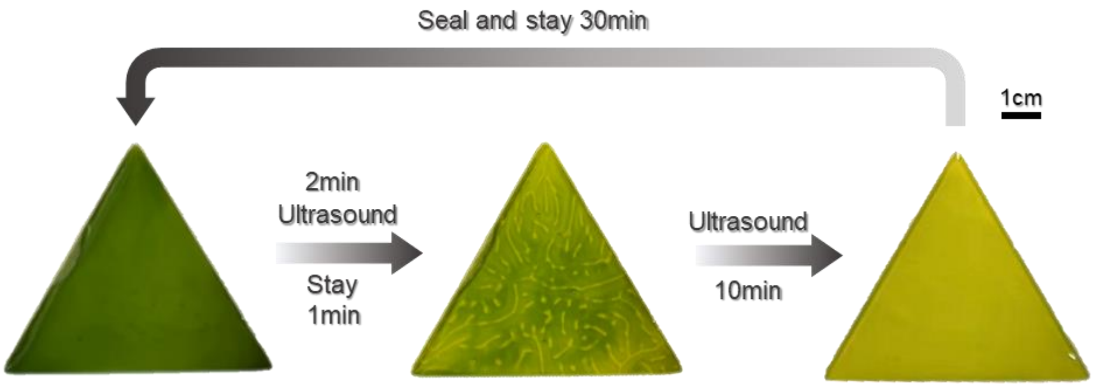

Supplementary Figure 49. Ultrasonically induced patterning of MV^{2+}/PN in a triangular mold containing PEG (5%) in buffer under daylight. Obtained by sonication for 150 s and stay for 60 s.

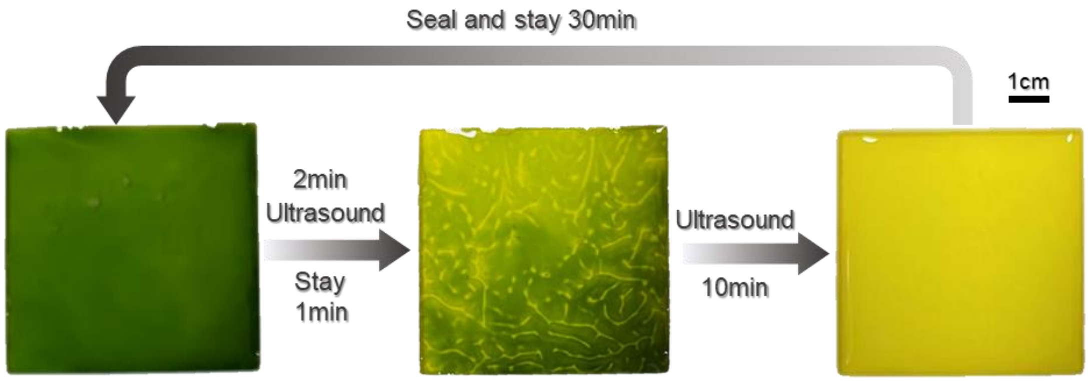

Supplementary Figure 50. Ultrasonically induced patterning of MV^{2+}/PN in a square mold containing PEG (5%) in buffer under daylight. Obtained by sonication for 150 s and stay for 60 s.

27. NMR spectrum.

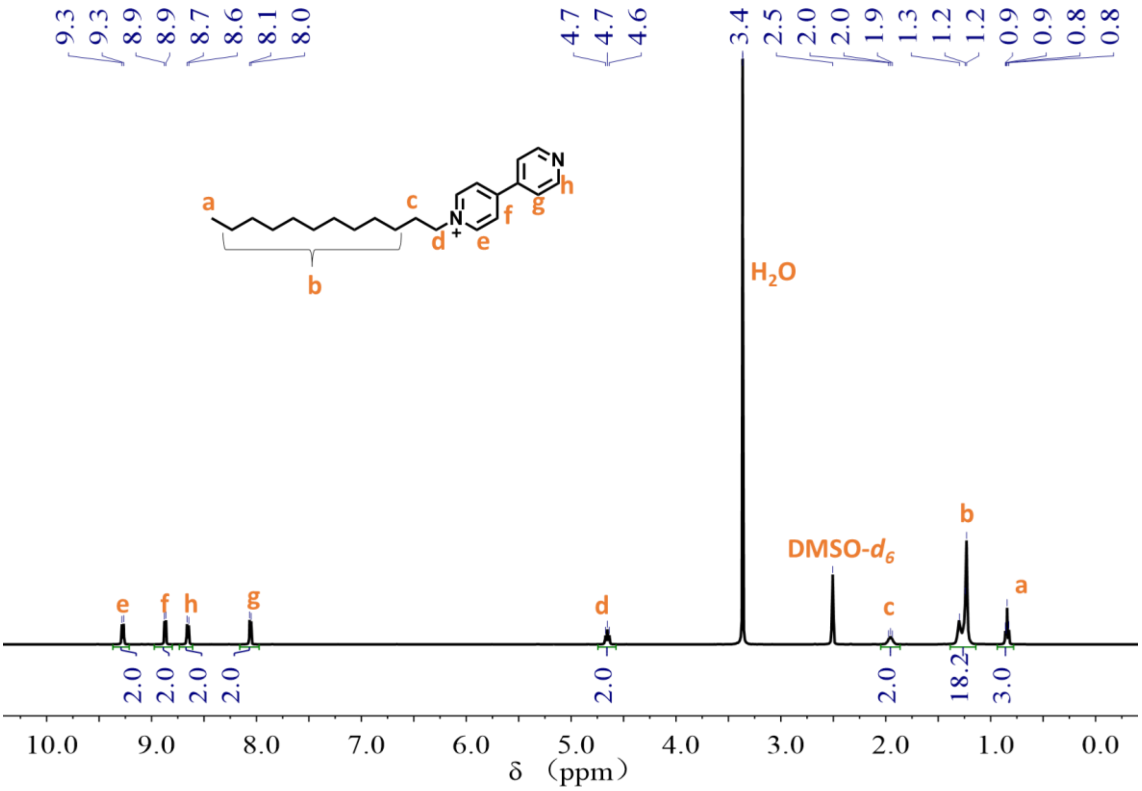

Supplementary Figure 51. ^{1}H-NMR spectrum of 1-dodecyl-1-ium in DMSO-d_6 (400 MHz).

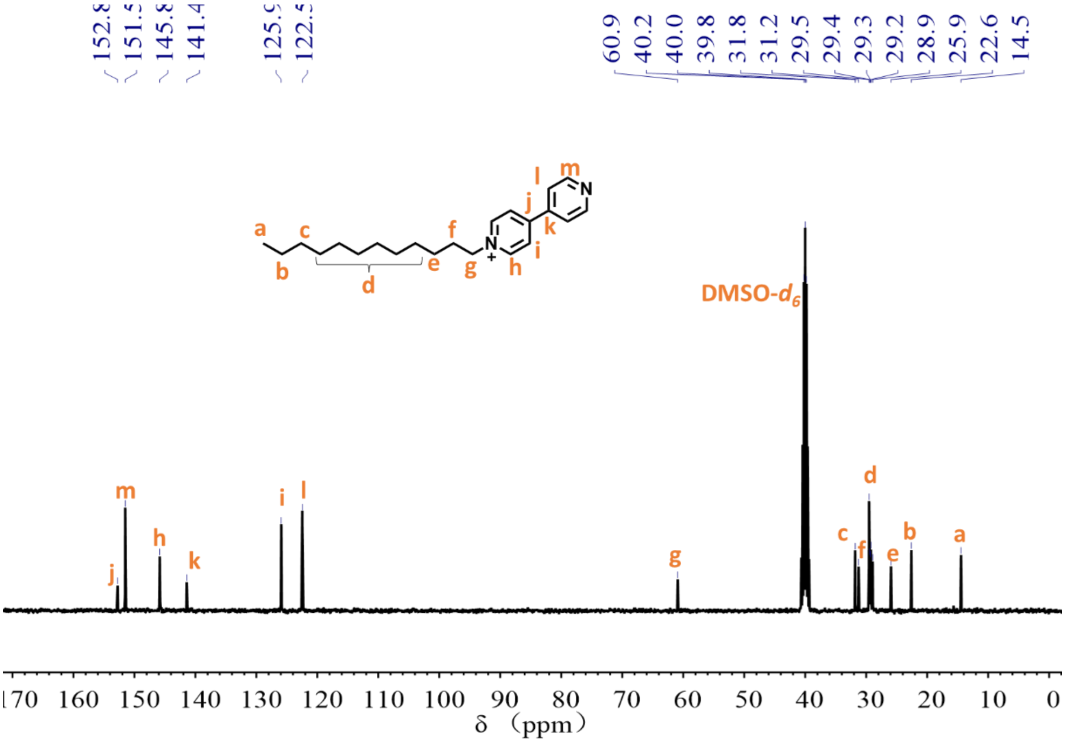

Supplementary Figure 52. ^{13}C-NMR spectrum of 1-dodecyl-[4,4'-bipyridin]-1-ium in DMSO-d_6.

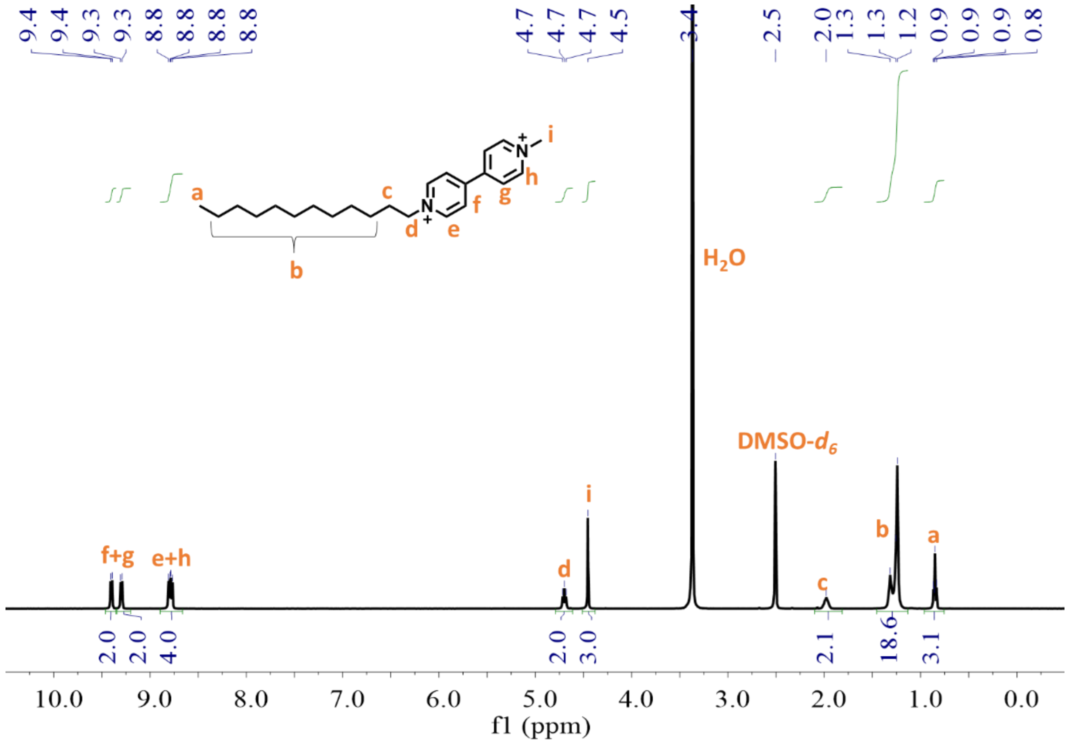

Supplementary Figure 53. ^{1}H-NMR spectrum of C_{12}-MV^{2+} in DMSO-d_6 (400 MHz).

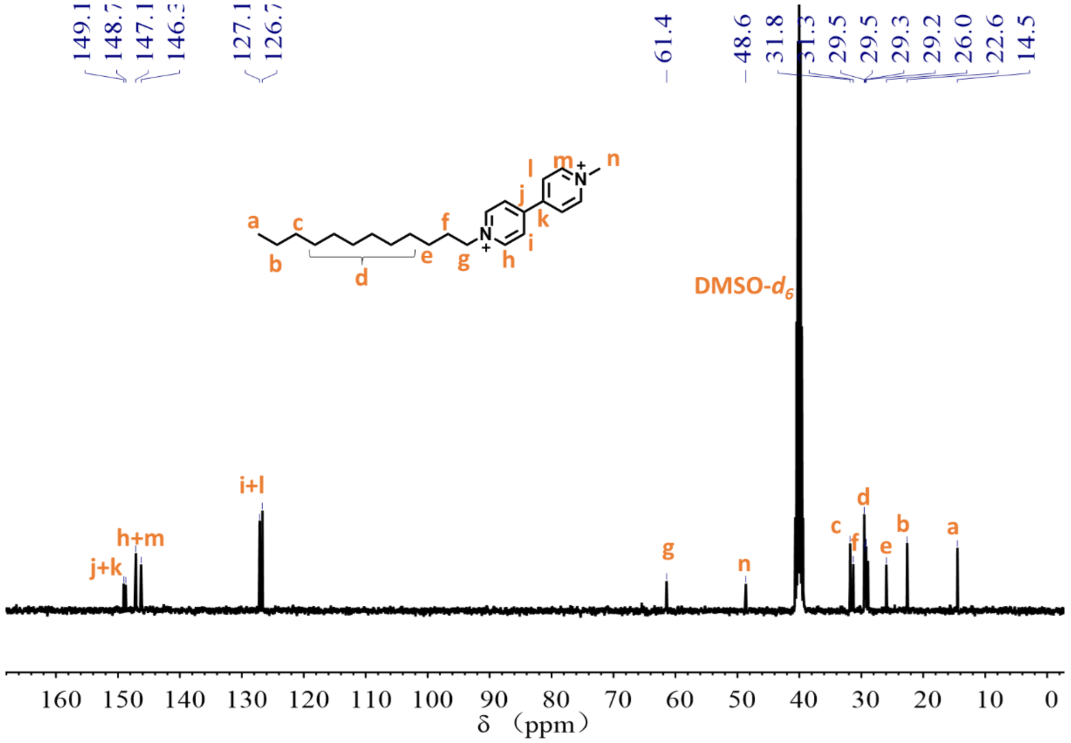

Supplementary Figure 54. ^{13}C-NMR spectrum of C_{12}-MV^{2+} in DMSO-d_6.

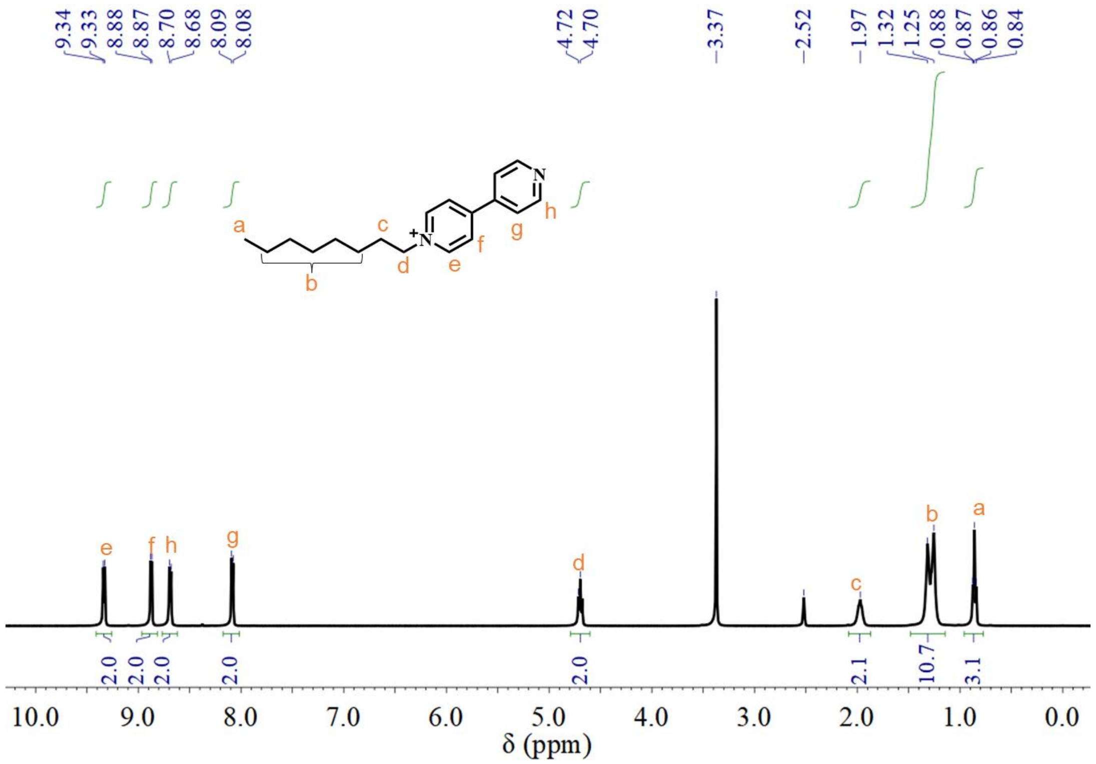

Supplementary Figure 55. ^{1}H-NMR spectrum of 2 in DMSO-d_6 (400 MHz).

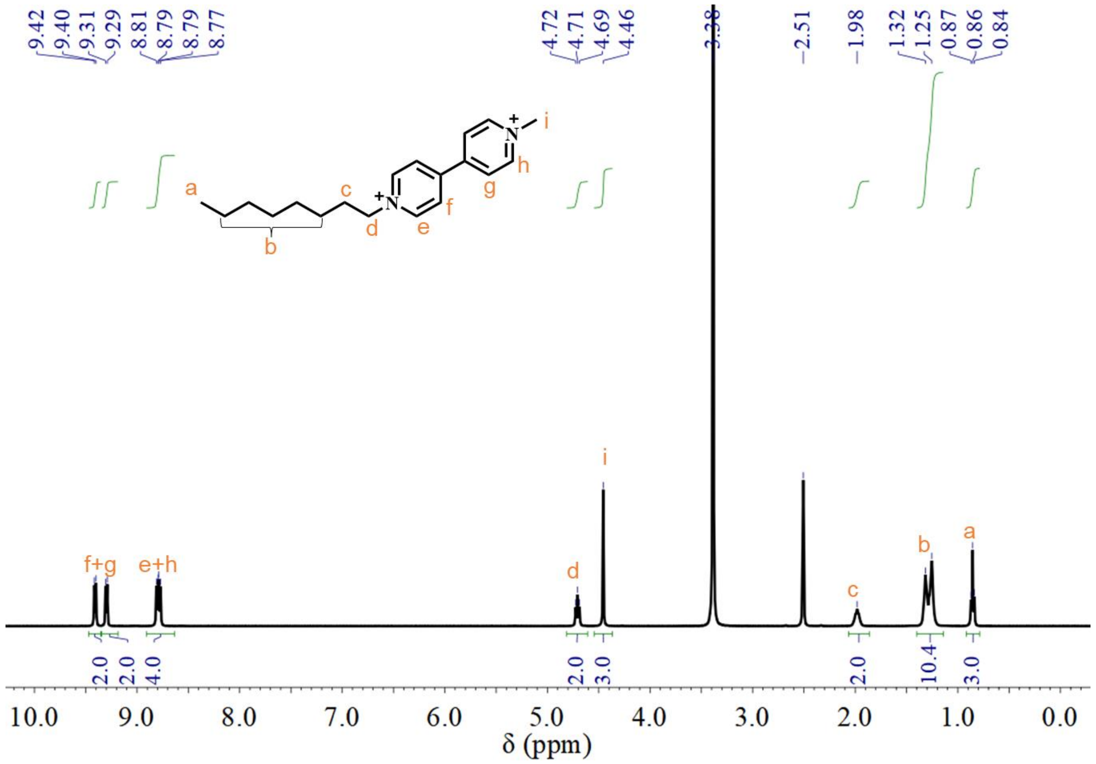

Supplementary Figure 56. ^{1}H-NMR spectrum of C_8-MV^{2+} in DMSO-d_6 (400 MHz).

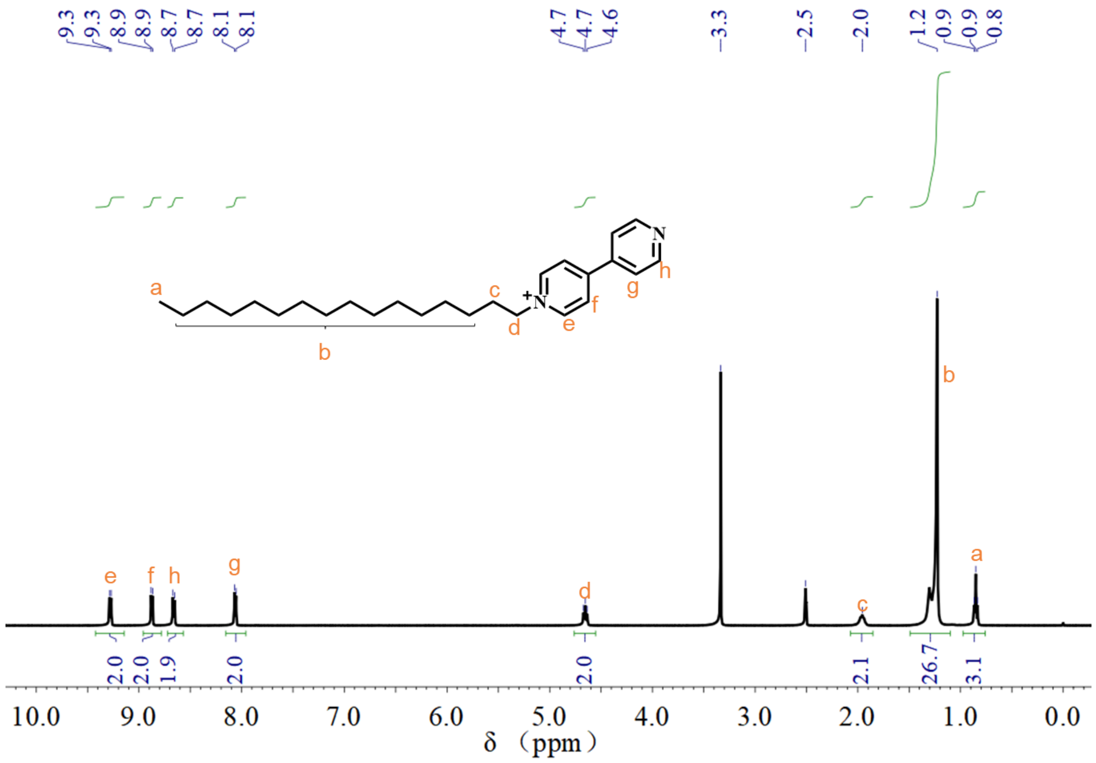

Supplementary Figure 57. ^{1}H-NMR spectrum of 3 in DMSO-d_6 (400 MHz).

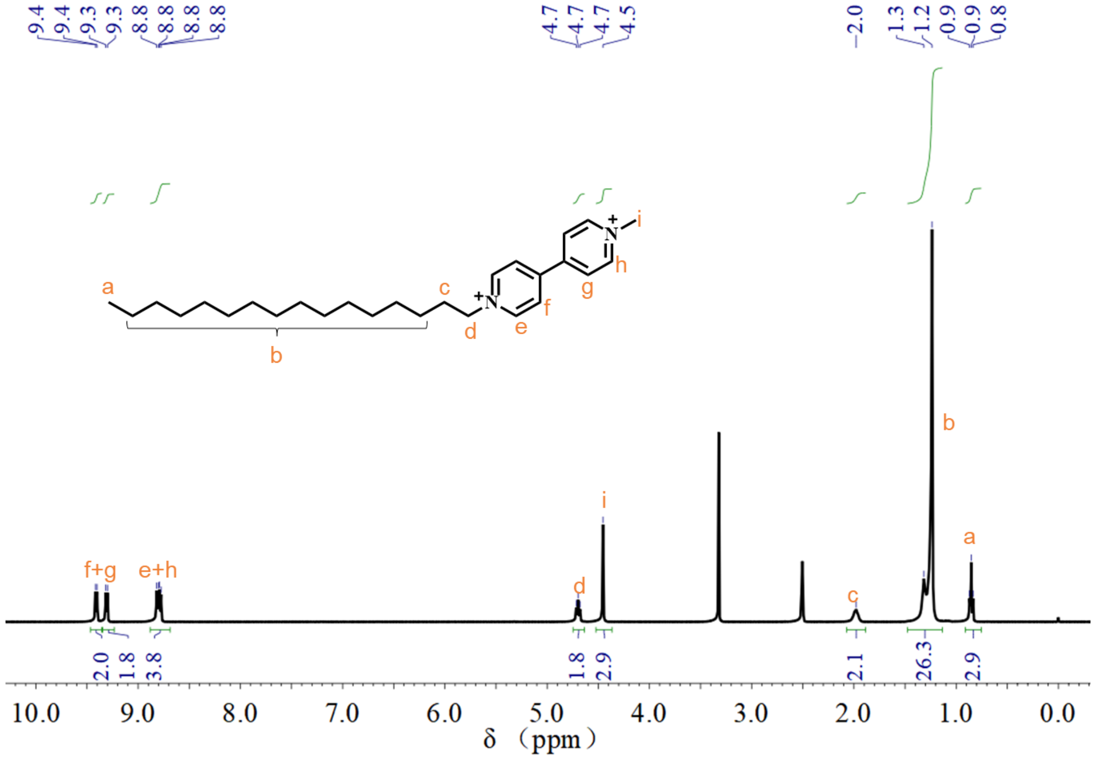

Supplementary Figure 58. ^{1}H-NMR spectrum of C_{16}-MV^{2+} in DMSO-d_6 (400 MHz).

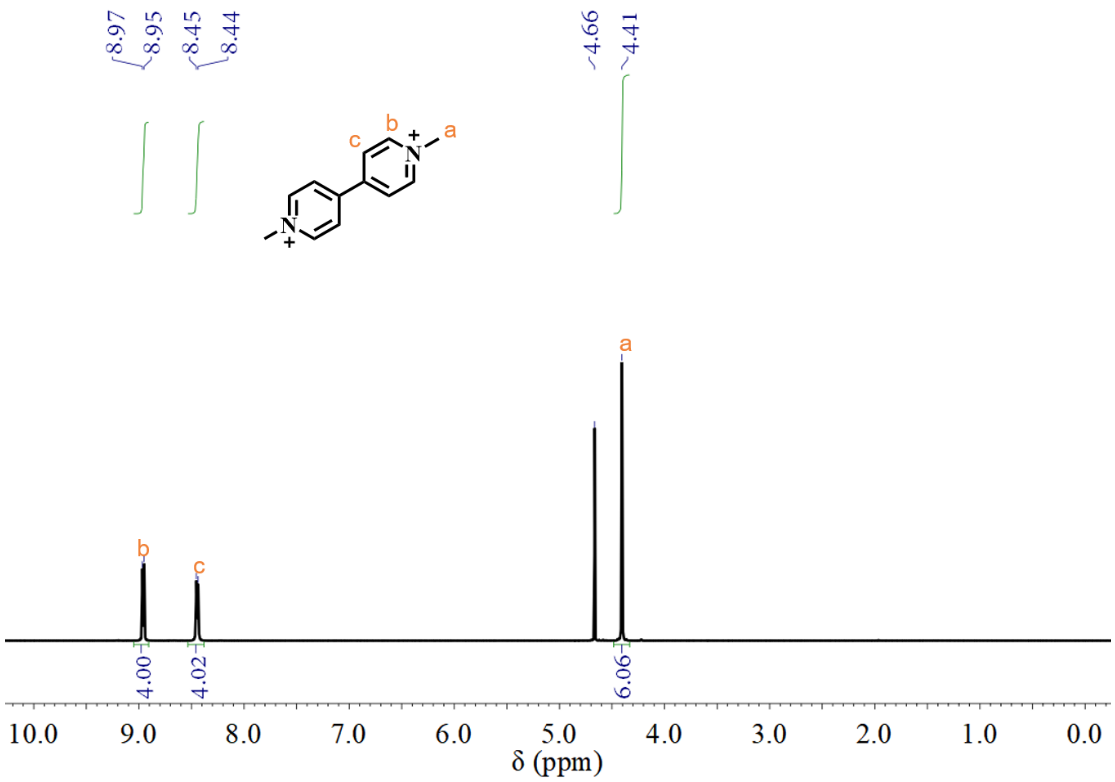

Supplementary Figure 59. ^{1}H-NMR spectrum of MV^{2+} in D_2O (400 MHz).

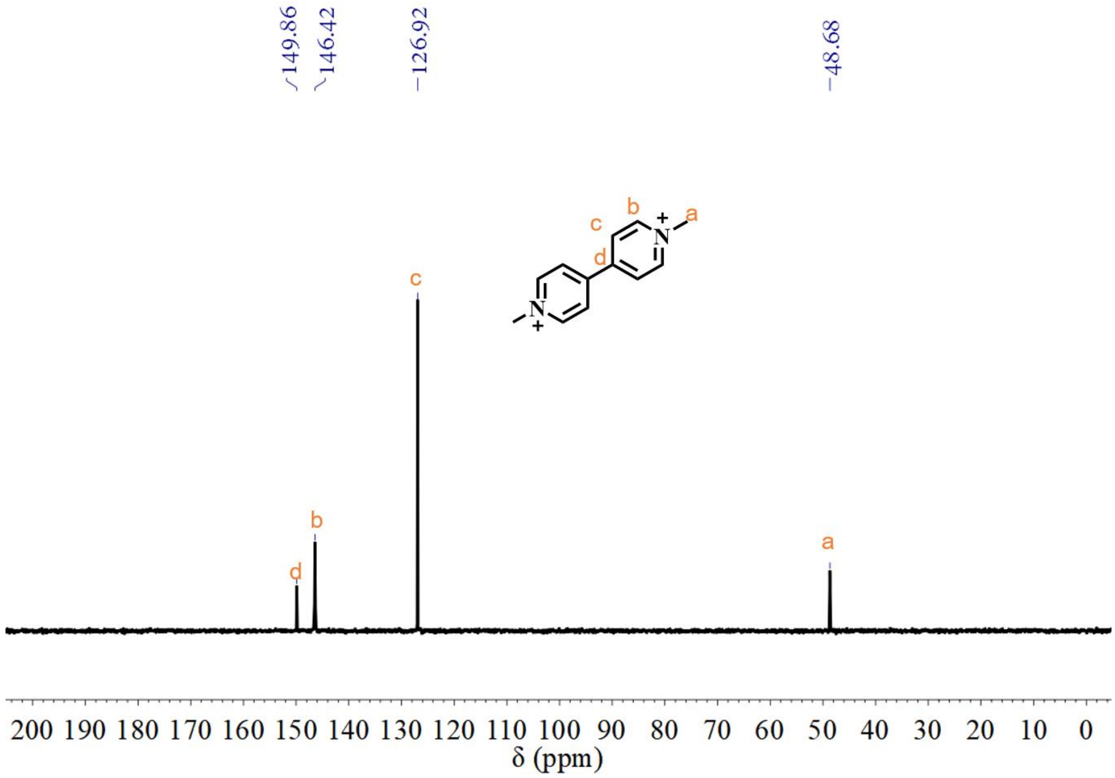

Supplementary Figure 60. ^{13}C-NMR spectrum of MV^{2+} in D_2O (400 MHz).

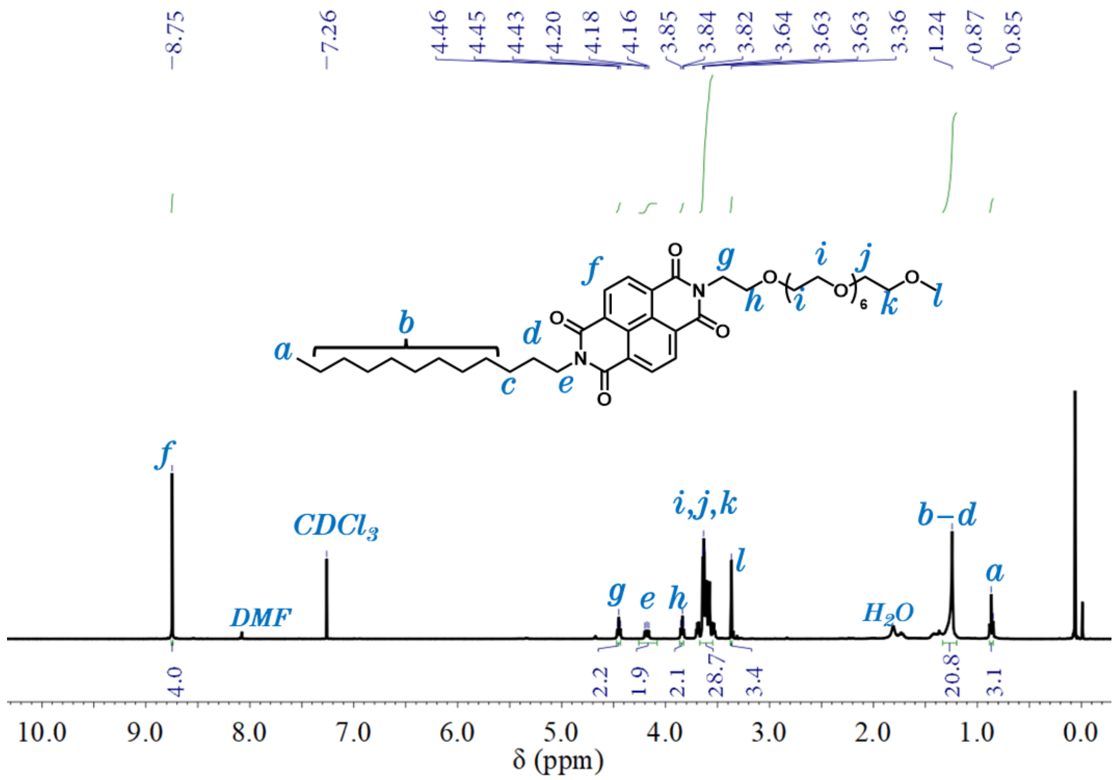

Supplementary Figure 61. ^{1}H-NMR spectrum of C_{12}-NDI-PEG350 in $CDCl_3$.

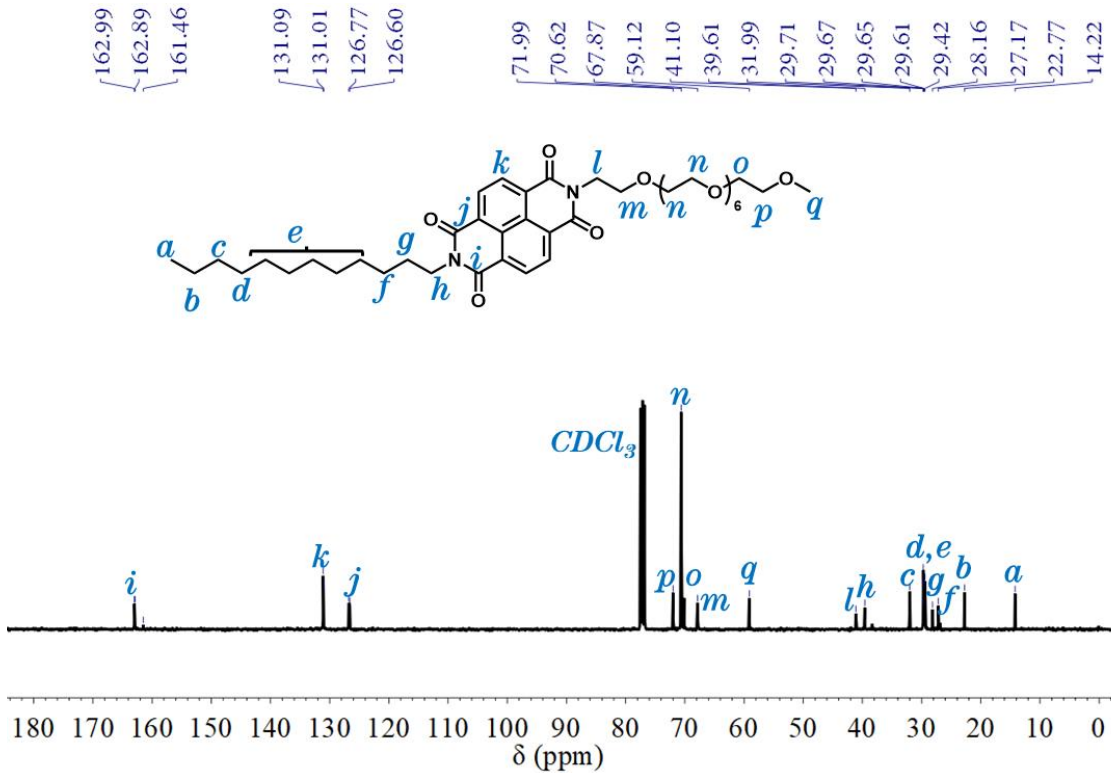

Supplementary Figure 62. ^{13}C-NMR spectrum of C_{12}-NDI-PEG350 in $CDCl_3$.

Reference

1. Zhang XA, et al. A Facile Synthetic Route to Functional Poly(phenylacetylene)s with Tunable Structures and Properties. Macromolecules 44, 6724-6737 (2011).
2. Oshchepkov, M. & Popov, K. in Desalination and Water Treatment. Ch. Chapter 15, (2018).
