# Supplementary material for: Mechanosensitive non-equilibrium supramolecular polymerization in closed chemical systems
Source: Nat Commun. 2023 May 29;14:3084. doi: 10.1038/s41467-023-38948-x (PMC10227035; doi:10.1038/s41467-023-38948-x)
Supplement: Supplementary file 3 — Description of Additional Supplementary Files [file 41467_2023_38948_MOESM3_ESM.pdf]

Description of Additional Supplementary Files

File Name: Supplementary Movie 1
Description: Shaking/staying induced discoloration of C12-MV2+

File Name: Supplementary Movie 2
Description: Shaking-induced fluorescence quenching of C12- MV2+/PN

File Name: Supplementary Movie 3
Description: Shaking-induced enhancement of fluorescence emission of MV2+/PN

File Name: Supplementary Movie 4
Description: Patterning of MV2+/PN under ultrasound and staying with 5%PEG under 365nm UV light

File Name: Supplementary Movie 5
Description: Patterning of MV2+/PN under ultrasound and staying with 5%PEG under daylight

File Name: Supplementary Movie 6
Description: Patterning of C12- MV2+ (2.5mM) under ultrasound and staying

File Name: Supplementary Movie 7
Description: Fluorescence patterning under continuous ultrasound of MV2+/PN with 5%PEG
